# Ack1 overexpression promotes metastasis and indicates poor prognosis of hepatocellular carcinoma

**DOI:** 10.18632/oncotarget.5872

**Published:** 2015-10-20

**Authors:** Xiong Lei, Yun-feng Li, Guo-dong Chen, Di-peng Ou, Xiao-xin Qiu, Chao-hui Zuo, Lian-Yue Yang

**Affiliations:** ^1^ Liver Cancer Laboratory, Xiangya Hospital, Central South University, Changsha 410008, Hunan, China; ^2^ Department of Surgery, Xiangya Hospital, Central South University, Changsha 410008, Hunan, China; ^3^ Department of Abdominal Surgical Oncology, Affiliated Cancer Hospital of Xiangya School of Medicine, Central South University, Changsha 410013, Hunan, China

**Keywords:** Ack1, hepatocellular carcinoma, prognosis, metastasis

## Abstract

Despite the substantial data supporting the oncogenic role of Ack1, the predictive value and biologic role of Ack1 in hepatocellular carcinoma (HCC) metastasis remains unknown. In this study, both correlations of Ack1 expression with prognosis of HCC, and the role of Ack1 in metastasis of HCC were investigated *in vitro* and *in vivo*. Our results showed that Ack1 was overexpressed in human HCC tissues and cell lines. High Ack1 expression was associated with HCC metastasis and determined as a significant and independent prognostic factor for HCC after liver resection. Ack1 promoted HCC invasion and metastasis *in vitro* and *in vivo*. Mechanistically, we confirmed that Ack1 enhanced invasion and metastasis of HCC via EMT by mediating AKT phosphorylation. In conclusion, our study shows Ack1 is a novel prognostic biomarker for HCC and promotes metastasis of HCC via EMT by activating AKT signaling.

## INTRODUCTION

Hepatocellular carcinoma (HCC) is the second leading cause of cancer-related death worldwide in men. [1] Long-term clinical results demonstrated by our team suggest that liver resection is still considered as the first choice and the most effective “curative” treatment for HCC, especially for solitary large HCC. [2] However, the overall prognosis of HCC is still far from satisfactory due to high incidences of tumor recurrence and metastasis, [2, 3] with a 5-year recurrence rate of approximately 60% after hepatic resection in our and other centers. [2, 4, 5] Therefore, It is important to further study the molecular mechanisms underlying metastasis of HCC and to research novel prognostic biomarkers for HCC.

We have previously identified a specific subtype of hepatocellular carcinoma, solitary large hepatocellular carcinoma (SLHCC, only one nodule, and tumor diameter >5 cm), with a lower rate of tumor recurrence and metastasis and a better long-term survival outcome, compared with nodular hepatocellular carcinoma (NHCC, tumor nodules ≥2). [4] Further clinical studies confirmed that the metastatic potential of SLHCC was comparable with small hepatocellular carcinoma (SHCC, ≤ 5 cm in diameter), but significantly lower than NHCC. [2, 4] To understand the molecular mechanisms underlying the difference of metastatic potential between SLHCC and NHCC, cDNA microarray analysis was used to identify difference of gene profile between these two subtypes of HCC. [6, 7] Among the 8464 human genes screened, 313 genes were up-regulated in NHCC than in SLHCC, including RhoC, [8–10] protocadherin LKC [6, 7] and Ack1.

Ack1, a nonreceptor tyrosine kinase, was originally identified as a Cdc42-interacting protein and a Cdc42 effector. [11, 12] Ack1 overexpression has been observed in multiple human cancers including prostate, gastric, and pancreatic cancers. [13–15] Substantial data indicate that Ack1 is implicated in metastatic behavior, cell spreading and migration. [16] Overexpression of Ack1 can increase the invasive phenotype of breast cancer cells [17] and mediate melanoma cell spreading. [18] These data indicate that Ack1 is involved in invasion and metastasis in HCC, yet its precise role in HCC remains unclear. Metastasis of tumors leads to a very poor prognosis for patients suffering from cancer. [3] We postulate Ack1 may be a poor prognostic biomarker and is associated with HCC metastasis.

In the present study, we determined Ack1 expression and its clinical significance of prognostic marker in HCC according to REMARK guidelines for reporting prognostic biomarkers in cancer. [19] We further identified the effect of Ack1 on metastasis of HCC and its underlying molecular mechanism by using a series of *in vitro* and *in vivo* assays.

## RESULTS

### Ack1 expression is significantly increased in HCC tissues

**Figure 1:**
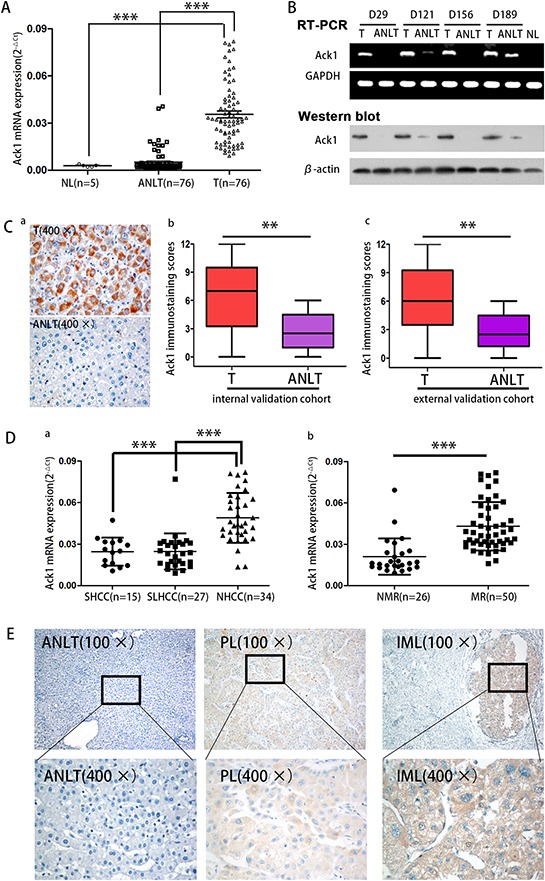
Ack1 expression is significantly increased in HCC and correlated with HCC metastasis **A.** Ack1 mRNA of tumor tissues in the training cohort is higher than that in ANLTs by quantitative real-time PCR (qRT-PCR). NL, ANLT, T represent normal liver tissue, adjacent nontumorous liver tissue, tumor tissue. **B.** Ack1 expression in paired T and ANLT in the training cohort was detected by semiquantitative RT-PCR and western blot. GAPDH and β-actin was used as an internal loading control. **C.** Immunohistochemical analysis of Ack1 expression in paired HCC tissues. Representative images (400 ×) of liver tissues with Ack1 protein staining were taken (Ca) and statistical analysis of expression in HCCs (Cb and Cc). **D.** a. Ack1 mRNA in NHCC with high metastatic potential expressed significantly higher than that in SHCC and SLHCC with relative low metastatic potential. b. The patients with metastasis and/or recurrence exhibited higher Ack1 mRNA expression in tumor tissues than those without. MR, NMR represents HCC with metastasis and/or recurrence, HCC without metastasis and/or recurrence respectively. **E.** A representative immunohistochemistry (IHC) staining of Ack1 protein in liver tissues. Intrahepatic metastasis lesion (IMLs) showed the highest level. ANLT, PL represents adjacent nontumorous liver tissue, primary lesion respectively. ** *P* < 0.01, *** *P* < 0.001.

Previous cDNA microarray analysis was performed to identify difference of gene profile between SLHCC and NHCC. [6, 7] Results showed that Ack1 expression was significantly higher in HCC tissues than in adjacent nontumorous liver tissues (ANLTs). Furthermore, Ack1 expression was also significantly higher in NHCC than in SLHCC. To confirm the results of cDNA microarray, quantitative real time-PCR (qRT-PCR) was performed to determine Ack1 expression in 76 HCCs of training cohort (Supplementary Figure S1 and Supplementary Table S1). Ack1 mRNA was up-regulated in HCC tumors compared with that in ANLTs (0.0347 ± 0.0187 *vs*. 0.0049 ± 0.0064, *P* < 0.0001, Figure 1A). Semiquantitative RT-PCR and western blot (Figure 1B) showed the similar results as qRT-PCR in these matched specimens. Immunohistochemical (IHC) assays of specimen from internal and external validation cohort (Supplementary Figure S1 and Supplementary Table S1) showed Ack1 expression was highly located in the cytoplasm (Figure 1Ca), and further validated that the expression level of Ack1 was significantly higher in HCC tumors than that in ANLTs (Figure 1Cb & 1Cc). Taken together, these data proved that the expression of Ack1 was up-regulated in HCC.

### Up-regulated Ack1 expression is significantly correlated with HCC metastasis

We further assessed Ack1 expression level among three different subtypes of HCC including SHCC, SLHCC and NHCC, Ack1 expression level was the highest in NHCC with the greatest metastatic potential and the poorest clinical outcome (*P* < 0.001; Figure 1Da). The patients with recurrence or metastasis of HCC exhibited higher Ack1 mRNA expression than those without recurrence or metastasis (0.0456 ± 0.0159 *vs*. 0.0179 ± 0.0057, *P* < 0.0001; Figure 1Db). In addition, IHC also showed the expression level of Ack1 was progressively increased from ANLTs, primary lesions (PLs) to intrahepatic metastasis lesions (IMLs) (Figure 1E). Above all, those results indicated that Ack1 was associated with HCC metastasis.

### Associations of high Ack1 expression with Clinicopathological characteristics

Next, we analyzed the association of clinicopathological variables with high Ack1 expression level. In training cohort, Mann-Whitney *U*-test showed Ack1 mRNA and protein expression was significantly associated with poor prognosis-related clinicopathological variables such as tumor number, tumor differentiation, vascular invasion, pathologic satellites nodules, and TNM stage (Table 1). These findings were further validated in internal validation cohort. Results showed high Ack1 expression was also significantly related to almost all previously known malignant clinicopathological variables such as tumor number, tumor differentiation, vascular invasion, pathologic satellite nodules, TNM stage (Table 2). In contrast, high Ack1 expression was not associated with gender, age, HBsAg, liver cirrhosis, AFP, TBil, albumin, tumor size, capsular formation, and BCLC stage in training cohort and internal validation cohort (Table 1, Table 2).

**Table 1:** Correlations between Ack1 expression and clinicopathological data of HCC in training cohort

Clinicopathologic variable	*n*	Ack1 expression		*P* Value	
		mRNA(×10^−2^)	Protein	mRNA	Protein
**Gender**					
Female	12	3.12 ± 0.21	0.77 ± 0.31		
Male	64	3.69 ± 0.17	0.72 ± 0.24	0.078	0.302
**Age (years)**					
≤60	45	3.06 ± 1.02	0.76 ± 0.53		
>60	31	3.78 ± 0.27	0.67 ± 0.34	0.107	0.062
**HBsAg**					
Positive	68	3.27 ± 0.43	0.70 ± 0.11		
Negative	8	3.88 ± 0.34	0.83 ± 0.26	0.091	0.076
**Liver cirrhosis**					
Presence	66	2.98 ± 0.85	0.86 ± 0.36		
Absence	10	3.82 ± 0.57	0.64 ± 0.14	0.132	0.082
**AFP**					
≤20 μg/L	20	3.29 ± 0.61	0.65 ± 0.32		
>20 μg/L	56	3.81 ± 1.07	0.88 ± 0.24	0.408	0.054
**TBil(μmol/L)**					
≤17.1	46	3.23 ± 0.92	0.71 ± 0.47		
>17.1	30	4.01 ± 0.28	0.82 ± 0.25	0.310	0.072
**Albumin(g/L)**					
≤35	38	3.38 ± 0.39	0.68 ± 0.23		
>35	38	3.56 ± 0.52	0.84 ± 0.09	0.308	0.089
**Tumor number**					
Solitary	52	2.56 ± 0.86	0.67 ± 0.81		
Multiple	24	4.67 ± 0.64	1.19 ± 0.36	**0.008**	**0.032**
**Tumor size**					
≤5 cm	42	3.27 ± 0.21	0.83 ± 0.41		
>5 cm	34	3.59 ± 0.17	0.89 ± 0.24	0.119	0.096
**Edmondson-Steiner grade**					
Low grade (I and II)	49	2.78 ± 0.93	0.69 ± 0.29		
High grade (III and IV)	27	4.49 ± 0.57	0.90 ± 0.72	**0.014**	**0.032**
**Vascular invasion**					
Presence	33	4.58 ± 0.21	0.89 ± 0.28		
Absence	43	3.12 ± 0.35	0.61 ± 0.19	**0.007**	**0.016**
**Satellite nodules**					
Presence	27	2.83 ± 0.74	0.60 ± 0.31		
Absence	49	4.91 ± 1.37	1.09 ± 0.24	**<0.001**	**0.009**
**Capsular formation**					
Presence	31	3.18 ± 0.21	0.69 ± 0.28		
Absence	45	3.24 ± 0.18	0.74 ± 0.16	0.302	0.103
**TNM Stage**					
I	32	2.97 ± 0.65	0.63 ± 0.19		
II–III	44	4.72 ± 0.73	0.92 ± 0.26	**0.021**	**0.015**
**BCLC Stage**					
0/A	28	3.60 ± 0.74	0.71 ± 0.72		
B/C	48	3.61 ± 1.37	0.73 ± 0.43	0.107	0.089

**Table 2:** Correlations between Ack1 expression and clinicopathological data of HCC in internal validation cohort

Clinicopathologic variable	*n*	Ack1 expression levels		*P* value
		Low	High	
**Gender**				
Female	23	11	12	
Male	118	42	76	0.268
**Age (years)**				
≤60	80	31	49	
>60	61	22	39	0.744
**HBsAg**				
Positive	130	48	82	
Negative	11	5	6	0.838
**Liver cirrhosis**				
Presence	122	45	77	
Absence	19	8	11	0.662
**AFP**				
≤20 μg/L	39	17	22	
>20 μg/L	102	36	66	0.363
**TBil(μmol/L)**				
≤17.1	84	29	55	
>17.1	57	24	33	0.362
**albumin(g/L)**				
≤35	69	26	43	
>35	72	27	45	0.982
**Tumor number**				
Solitary	98	46	52	
Multiple	43	7	36	**0.001**
**Tumor size**				
≤5 cm	79	34	45	
>5 cm	62	19	43	0.132
**Edmondson-Steiner grade**				
Low grade (I and II)	92	45	47	
High grade (III and IV)	49	8	41	**<0.001**
**Vascular invasion**				
Presence	81	20	61	
Absence	60	33	27	**<0.001**
**Satellite nodules**				
Presence	52	12	40	
Absence	89	41	48	**0.007**
**Capsular formation**				
Presence	56	18	38	
Absence	85	35	50	0.279
**TNM Stage**				
I	54	31	23	
II–III	87	22	65	**0.042**
**BCLC Stage**				
0/A	51	16	35	
B/C	90	37	53	0.251

### High Ack1 expression predicts poor prognosis in HCC patients

**Figure 2:**
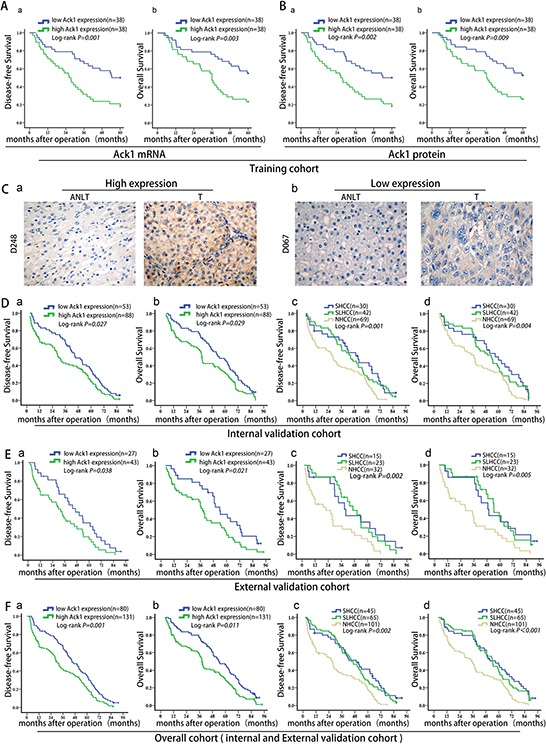
Ack1 is a potential prognostic predictor of poor clinical outcome after liver resection **A. & B.** Disease-free survival (Aa, Ba) and overall survival (Ab, Bb) of HCC patients in the training cohort classified into low- and high-Ack1 groups based on median of Ack1 mRNA and protein expression level. **C.** Representative images of HCC tissues with different Ack1 protein staining. According to IHC immunostaining score, HCC patients dichotomized into high Ack1 expression group (> 3) (Ca) and low Ack1 expression group (≤ 3) (Cb). **D. & E.** disease-free survival (Da, Ea) and overall survival (Db, Eb) in the internal (*n* = 141) and external (*n* = 70) validation cohorts. Survival analysis of disease-free survival (Dc, Ec) and overall survival (Dd, Ed) of three subtypes of HCC (SHCC, SLHCC and NHCC). **F.** Disease-free survival (Fa, Fc) and overall survival (Fb, Fd) of HCC patients in the overall cohort (*n* = 211) integrated by internal (*n* = 141) and external (*n* = 70) validation cohorts.

As metastasis is the main causative factor for HCC poor outcome, we further interrogated whether Ack1 expression could predict prognosis of HCC patients according to REMARK guidelines for reporting prognostic biomarkers in cancer. [19] In training cohort, patients were classified into low- and high-Ack1 groups based on median of Ack1 mRNA and protein expression level. Kaplan-Meier analysis showed HCC patients with high Ack1 mRNA expression had poorer disease-free survival (*P* = 0.001, Figure 2Aa) and overall survival (*P* = 0.003, Figure 2Ab) than patients with low Ack1 mRNA expression. What's more, a similar significant difference in disease-free survival (DFS) (*P* = 0.002, Figure 2Ba) and overall survival (OS) (*P* = 0.009, Figure 2Bb) was also obtained based on Ack1 protein expression level. In addition, high Ack1 expression was associated with high early tumor recurrence (within 2 years) rate according to Ack1 mRNA expression (*P* = 0.034; Supplementary Figure 2Aa) and Ack1 protein expression level (*P* = 0.025; Supplementary Figure 2Ab). Thus, we inferred that Ack1 was a potential biomarker to predict survival in addition to metastasis.

To estimate the accuracy of Ack1 to predict prognosis, data from the internal validation cohort (Supplementary Figure S1 and Supplementary Table S1) were further calculated. We found that Ack1 was highly expressed in 88 (88/141, 62.4%) cancer samples (Table 2), with no to weak expression in ANLTs (Figure 2C). Data showed that patients with high Ack1 expression had lower DFS (*P* = 0.027; Figure 2Da), lower OS (*P* = 0.029; Figure 2Db) and a significantly higher early recurrence rate (*P* = 0.035; Supplementary Figure S2B) than patients with low Ack1 expression. In line with these results, SHCC and SLHCC had higher DFS (*P* = 0.001; Figure 2Dc) and OS (*P* = 0.004; Figure 2Dd) rates than NHCC. Furthermore, univariable and multivariable analysis by Cox proportional hazards regression model in the internal validation cohort revealed that along with multiple nodules, higher Edmondson-Stainer grade, vascular invasion, satellite nodules, capsular formation, TNM stage and BCLC stage, high Ack1 expression was also an significant and independent prognostic factor for both DFS (HR 3.596; 95% CI 1.108 to 6.230, *P* = 0.012; Table 3) and OS (HR 2.306, 95% CI 1.178 to 7.203, *P* = 0.034; Table 4).

**Table 3:** Univariable and multivariable analysis of disease-free survival (DFS) and clinicopathologic variables of HCC in internal validation cohort

Clinicopathologic variables	*n*	Univariable analysis		Multivariable analysis	
		HR* (95% CI)	*P*	HR* (95% CI)	*P*
**Gender**					
Female	23	Reference			
Male	118	1.090(0.646 − 1.839)	0.748		NA
**Age (years)**					
≤60	80	Reference			
>60	61	1.271(0.855 − 1.889)	0.236		NA
**HBsAg**					
Positive	130	Reference			
Negative	11	0.871(0.502 − 1.514)	0.625		NA
**Liver cirrhosis**					
Absence	122	Reference		Reference	
Presence	19	1.456(0.964 − 2.198)	0.074	1.450(0.941−2.235)	0.092
**AFP**					
≤20 μg/L	39	Reference			
>20 μg/L	102	1.223(0.811 − 1.845)	0.336		NA
**TBil(μmol/L)**					
≤17.1	84	Reference			
>17.1	57	1.042(0.752 − 1.693)	0.171		NA
**albumin(g/L)**					
≤35	69	Reference			
>35	72	1.302(0.872 − 1.584)	0.106		NA
**Tumor number**					
Solitary	98	Reference		Reference	
Multiple	43	2.277(1.616 − 3.207)	**0.003**	6.000(2.627−13.706)	**0.009**
**Tumor size**					
≤5 cm	79	Reference		Reference	
>5 cm	62	1.130(0.819 − 1.479)	0.073	1.053(0.795−1.123)	0.081
**Edmondson-Steiner grade**					
Low grade (I and II)	92	Reference		Reference	
High grade (III and IV)	49	1.589(1.106 − 2.826)	**0.023**	1.408 (1.318−2.392)	**0.016**
**Vascular invasion**					
Absence	60	Reference		Reference	
Presence	81	1.630(1.128 − 2.355)	**0.009**	1.767(1.192−2.621)	**0.005**
**Satellite nodules**					
Absence	89	Reference		Reference	
Presence	52	1.149(1.134 − 2.026)	**0.027**	1.327(1.097−1.843)	**0.039**
**Capsular formation**					
Presence	56	Reference		Reference	
Absence	85	1.077(1.044−2.092)	**0.028**	1.030(0.865−1.722)	0.096
**TNM Stage**					
I	54	Reference		Reference	
II–III	87	1.922(1.345−2.766)	**<0.001**	1.651(1.112−2.275)	**0.003**
**BCLC Stage**					
0/A	51	Reference		Reference	
B/C	90	3.402(1.368−4.168)	**0.017**	4.020(3.619−7.823)	**0.036**
**Ack1 expression**					
Low	53	Reference		Reference	
High	88	3.676(1.176−7.389)	**0.004**	3.596(1.108−6.230)	**0.012**

* HR: hazard ratio

**Table 4:** Univariable and multivariable analysis of overall survival (OS) and clinicopathologic variables of HCC in internal validation cohort

Clinicopathologic variables	*n*	Univariable analysis		Multivariable analysis	
		HR* (95% CI)	*P*	HR* (95% CI)	*P*
**Gender**					
Female	23	Reference			
Male	118	1.076(0.706−1.638)	0.548		NA
**Age (years)**					
≤60	80	Reference			
>60	61	1.072(0.678−1.088)	0.146		NA
**HBsAg**					
Positive	130	Reference			
Negative	11	0.780(0.613−1.406)	0.540		NA
**Liver cirrhosis**					
Absence	122	Reference		Reference	
Presence	19	1.348(0.802−1.890)	0.082	1.020(0.804−1.624)	0.106
**AFP**					
≤20 μg/L	39	Reference		Reference	
>20 μg/L	102	1.064(0.830−1.754)	0.072	1.031(0.905−1.462)	0.185
**TBil(μmol/L)**					
≤17.1	84	Reference			
>17.1	57	1.107(0.915−2.051)	0.105		NA
**albumin(g/L)**					
≤35	69	Reference			
>35	72	0.874(0.879−1.454)	0.217		NA
**Tumor number**					
Solitary	98	Reference		Reference	
Multiple	43	1.167(1.068−4.227)	**0.006**	3.020(2.802−10.802)	**0.009**
**Tumor size**					
≤5 cm	79	Reference		Reference	
>5 cm	62	1.120(1.096−3.407)	**0.036**	1.064(0.705−2.048)	0.073
**Edmondson − Steiner grade**					
Low grade (I and II)	92	Reference		Reference	
High grade (III and IV)	49	1.487(1.140−2.012)	**0.032**	1.360(1.204−1.894)	**0.003**
**Vascular invasion**					
Absence	60	Reference		Reference	
Presence	81	1.402(1.382−3.043)	**0.026**	1.605(1.402−2.716)	**0.037**
**Satellite nodules**					
Absence	89	Reference		Reference	
Presence	52	1.316(1.241−1.972)	**0.021**	1.212(1.173−1.621)	**0.008**
**Capsular formation**					
Presence	56	Reference		Reference	
Absence	85	1.304(1.262−1.608)	**0.044**	1.802(1.506−6.180)	**0.017**
**TNM Stage**					
I	54	Reference		Reference	
II−III	87	1.702(1.455−3.066)	**<0.001**	1.705(1.480−3.064)	**0.001**
**BCLC Stage**					
0/A	51	Reference		Reference	
B/C	90	1.322(1.276−3.184)	**0.043**	3.210(2.016−6.749)	**0.013**
**Ack1 expression**					
Low	53	Reference		Reference	
High	88	2.547(1.297−4.819)	**0.014**	2.306(1.178−7.203)	**0.034**

* HR: hazard ratio

### External validation of the HCC prognostic significance of Ack1

Prognostic significance of Ack1 was further evaluated in an independent of 70 radically resected HCCs from external validation cohort (Supplementary Figure S1 and Supplementary Table S1). Data of the external validation cohort also generated from formaldehyde-fixed, paraffin-embedded paired HCC tissues by IHC. Unlike internal validation, we generated prognosis prediction blind to survival data. A total of 43 patients (43/70, 61.4%) had high Ack1 expression, and this was significantly associated with DFS (*P* = 0.038, Figure 2Ea), OS (*P* = 0.021, Figure 2Eb) and early recurrence rate (*P* = 0.037, Supplementary Figure S2C). In the same cohort, SHCC and SLHCC had higher DFS (*P* = 0.002, Figure 2Ec) and OS (*P* = 0.005, Figure 2Ed) rates than NHCC. High Ack1 expression was significantly related to the clinicopathological characteristics such as tumor number, tumor differentiation, vascular invasion, pathologic satellite nodules, and TNM stage (Supplementary Table S2). Univariable and multivariable analysis by Cox proportional hazards regression model showed that high Ack1 expression was still an independent prognostic factor for both DFS (HR 3.954, 95% CI 2.081 to 6.740, *P* = 0.022; Supplementary Table S3) and OS (HR 2.461, 95% CI 1.921 to 4.036, *P* = 0.001; Supplementary Table S4).

Finally, we integrated the internal and external validation cohort into the overall cohort to re-analyze the prognostic significance of Ack1. High Ack1 expression also could predict low DFS (*P* = 0.001, Figure 2Fa), low OS (*P* = 0.011, Figure 2Fb) and high early recurrence rate (*P* = 0.003, Supplementary Figure 2D). Similarly, SHCC and SLHCC had higher DFS (*P* = 0.002, Figure 2Fc) and OS (*P* < 0.001, Figure 2Fd) rates than NHCC. These results showed the validity of Ack1 to predict HCC prognosis in addition to metastasis.

### Ack1 enhances invasion and metastasis of HCC *in Vitro and in Vivo*

**Figure 3:**
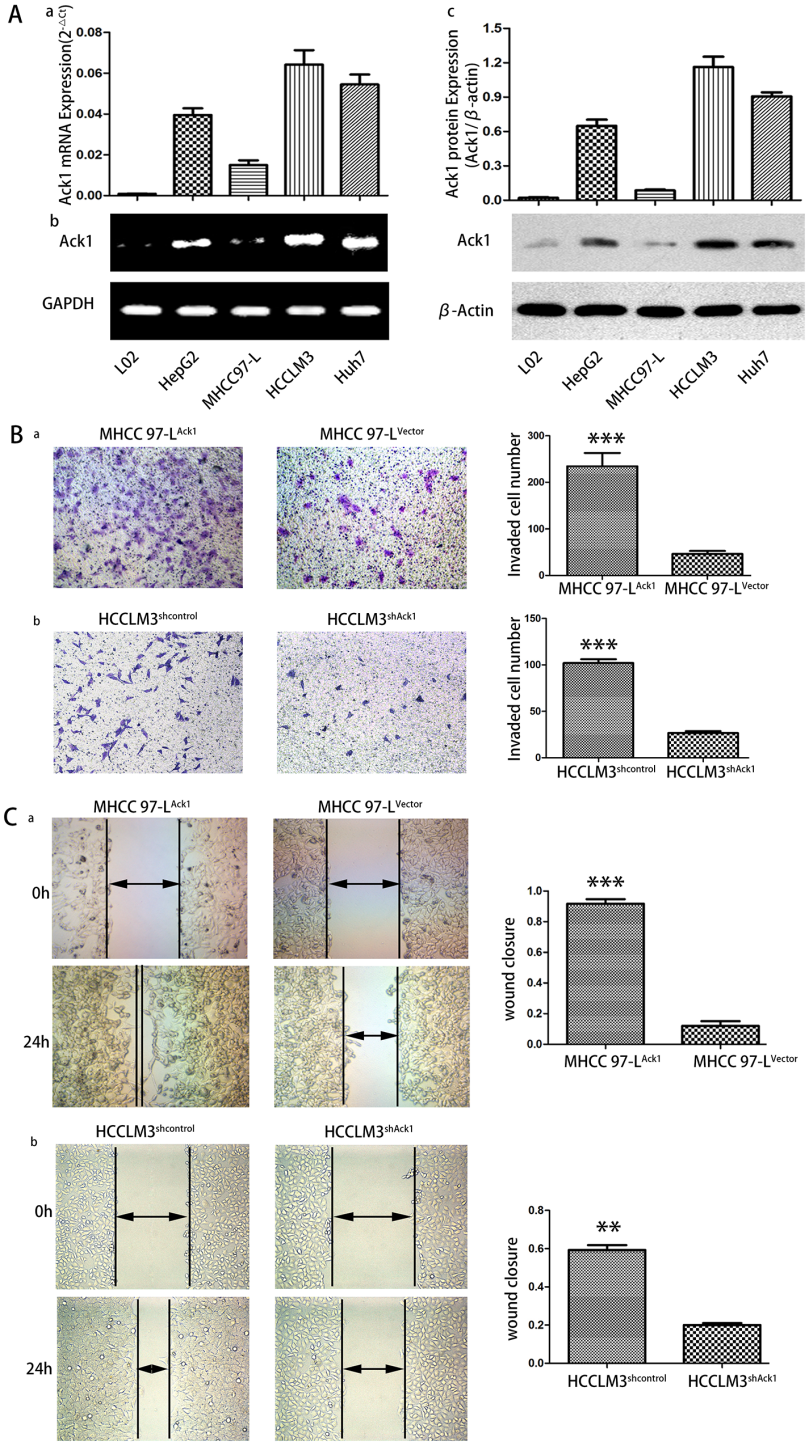
Ack1 promotes HCC cells invasion *in vitro* **A.** qRT-PCR analysis (a) and semiquantitative RT-PCR (b) were used to examine the expression of Ack1 in four HCC cell lines of HepG2, MHCC97-L, HCCLM3, Huh7 and normal liver cell lines L02. The levels of Ack1 mRNA expression in HCCLM3 exceed those in all other HCC cell lines. GAPDH was used as an internal loading control. (c) Analyzed by western blot, Ack1 protein expression was highest in HCCLM3 cells as compared with all other HCC cell lines. β-actin was used as the internal loading control. **B.** Boyden chamber invasion and **C.** wound-healing assays of MHCC97-L cells with Ack1 overexpression (MHCC97-L^Ack1^) or vector control (MHCC97-L^Vector^), and HCCLM3 cells with Ack1 knockdown (HCCLM3^shAck1^) or control (HCCLM3^shcontrol^). *** *P* < 0.001; ** *P* < 0.01.

To study the biological significance of Ack1 in HCC invasion and metastasis, the expression levels of Ack1 in normal liver cell lines L02 and four HCC cell lines with various metastatic capability, including HepG2, MHCC97-L and HCCLM3, Huh7, were measured (Figure 3A). Compared with normal liver cell lines L02, all HCC cell lines exhibited higher Ack1 expression detected by quantitative RT-PCR (Figure 3Aa), semiquantitative RT-PCR (Figure 3Ab) and western blot (Figure 3Ac). Meaningfully, Ack1 expression exhibited the highest in HCCLM3 with the greatest metastatic potential, [20, 21] consistent with results as determined in HCC samples.

To determine Ack1 function in HCC invasion and metastasis, we overexpressed Ack1 in MHCC97-L (named MHCC97-L^Ack1^, Supplementary Figure S3A) and knocked down Ack1 in HCCLM3 cells (named HCCLM3^shAck1^, Supplementary Figure S3B). As they have relatively low Ack1 expression and high Ack1 expression (Figure 3A), respectively and are adequate cell lines for studying metastatic cell model. [20, 21] We examined the effects of Ack1 on metastasis *in vitro* by transwell assays and wound-healing assays. In comparison with control cells, it was noted that Ack1 overexpression significantly increased MHCC97-L invasive ability by transwell assays (*P* < 0.001; Figure 3Ba) and enhanced motility of MHCC97-L cells by wound healing assay (*P* < 0.001, Figure 3Ca). Conversely, knockdown of Ack1 expression in HCCLM3 cells decreased cell invasion (*P* < 0.001; Figure 3Bb) and motility (*P* < 0.01; Figure 3Cb). Intriguingly, it was observed that overexpression of Ack1 induced the reorganization of actin cytoskeleton of MHCC97-L cells and changed the cell appearance to more spindle-like, fibroblastic morphology and extended more protrusions at the cell edges (Supplementary Figure S3C). On the contrary, knockdown of Ack1 resulted in disappearance of the stress fiber-like structures in HCCLM3 cells and changed the cell appearance to more cobble-stone morphology and extended less protrusions at the cell edges (Supplementary Figure S3D). An effect of Ack1 on the growth was identified. Cell proliferation assays and colony formation assays showed that overexpression of Ack1 increased cell proliferation of MHCC97-L cells (Supplementary Figure S4A, S4B), whereas knockdown of Ack1 decreased cell proliferation of HCCLM3 cells (Supplementary Figure S4C, S4D). These results support that Ack1 can enhance malignant phenotypes of HCC cells *in vitro*.

**Figure 4:**
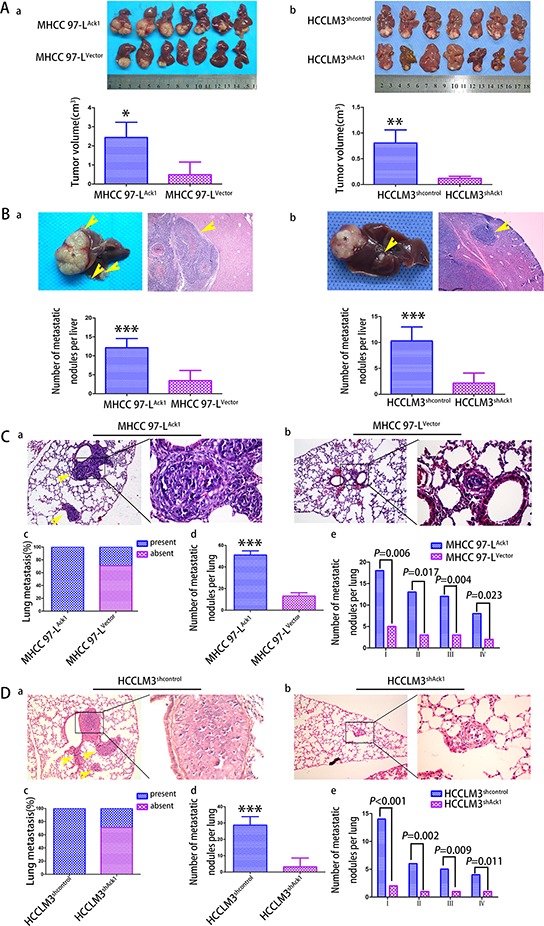
Ack1 promotes HCC cells metastasis *in vivo* **A.** The *in situ* HCC mouse model was constructed by using MHCC97-L cells with Ack1 overexpression (MHCC97-L^Ack1^) or vector control (MHCC97-L^Vector^) (Aa) and HCCLM3 cells with Ack1 knockdown (HCCLM3^shAck1^) or control (HCCLM3^shcontrol^) (Ab). The average size of *in situ* liver tumors in the Ack1 overexpression group (MHCC97-L^Ack1^) was dramatically larger than that of control group (MHCC97-L^Vector^), whereas the average size of liver tumor in Ack1 knockdown group (HCCLM3^shAck1^) was dramatically smaller than control (HCCLM3^shcontrol^). **B.** Representative pictures of intrahepatic metastasis from MHCC97-L cells with Ack1 overexpression (MHCC97-L^Ack1^) group (Ba) and HCCLM3 cells control (HCCLM3^shcontrol^) group (Bb). An asterisk marked the tumor developed at the implanted site, and yellow arrows marked intrahepatic metastatic lesions in the unimplanted site. Ack1 overexpression significantly increased the number of intrahepatic metastatic nodules, whereas Ack1 knockdown significantly decreased the number of intrahepatic metastatic nodules. **C, D.** (a and b) Representative micrographs of pulmonary metastasis. (c) The rate of nude mice with or without lung metastatic was calculated and compared. The number of total lung metastatic nodules (d) and lung metastatic nodules of each grade (e) per lung was analyzed between different groups in MHCC-97L cells (C) and HCCLM3 cells (D) H and E stain, original magnification: left, 100 × ; right, 400 ×. * *P* < 0.05; ** *P* < 0.01; *** *P* < 0.001.

To further verify the *in vitro* results, we evaluated the role of Ack1 by using an *in situ* mouse metastasis model *in vivo*. We observed that Ack1-overexpresed MHCC97-L cells MHCC97-L^Ack1^ formed dramatically larger size of *in situ* liver tumors than control MHCC97-L^Vector^ cells (*P* = 0.014; Figure 4Aa). More importantly, as compared with MHCC97-L^Vector^ group, MHCC97-L^Ack1^ group showed a dramatic increase in intrahepatic metastasis nodules (*P* < 0.001; Figure 4Ba), pulmonary metastasis rate (7/7(100.0%) *vs* 2/7(28.6%), *P* = 0.026; Figure 4Ca & 4Cb & 4Cc), pulmonary metastatic nodules (51 ± 6.7 *vs*. 13 ± 2.7, respectively, *P* < 0.001; Figure 4Ca & 4Cb & 4Cd). Similarly, the number of lung metastatic nodules of each grade was also greater in the MHCC97-L^Ack1^ group (Figure 4Ce). However, compared with HCCLM3^shcontrol^ group, knockdown of Ack1 in HCCLM3 cells significantly decreased tumor growth (*P* = 0.004; Figure 4Ab), intrahepatic metastasis nodules (*P* < 0.001; Figure 4Bb), pulmonary metastasis rate (2/7(28.6%) *vs* 7/7(100.0%), *P* = 0.026; Figure 4Da & 4Db & 4Dc), pulmonary metastatic nodules (6 ± 1.3 *vs*. 29 ± 3.2, respectively, *P* < 0.001; Figure 4Da & 4Db & 4Dd). Similarly, the number of lung metastatic nodules of each grade was also reduced (Figure 4De). All together, these data demonstrate that Ack1 can enhance metastatic potential of HCC *in vivo*.

### Metastasis is promoted by Ack1 via facilitating emt

**Figure 5:**
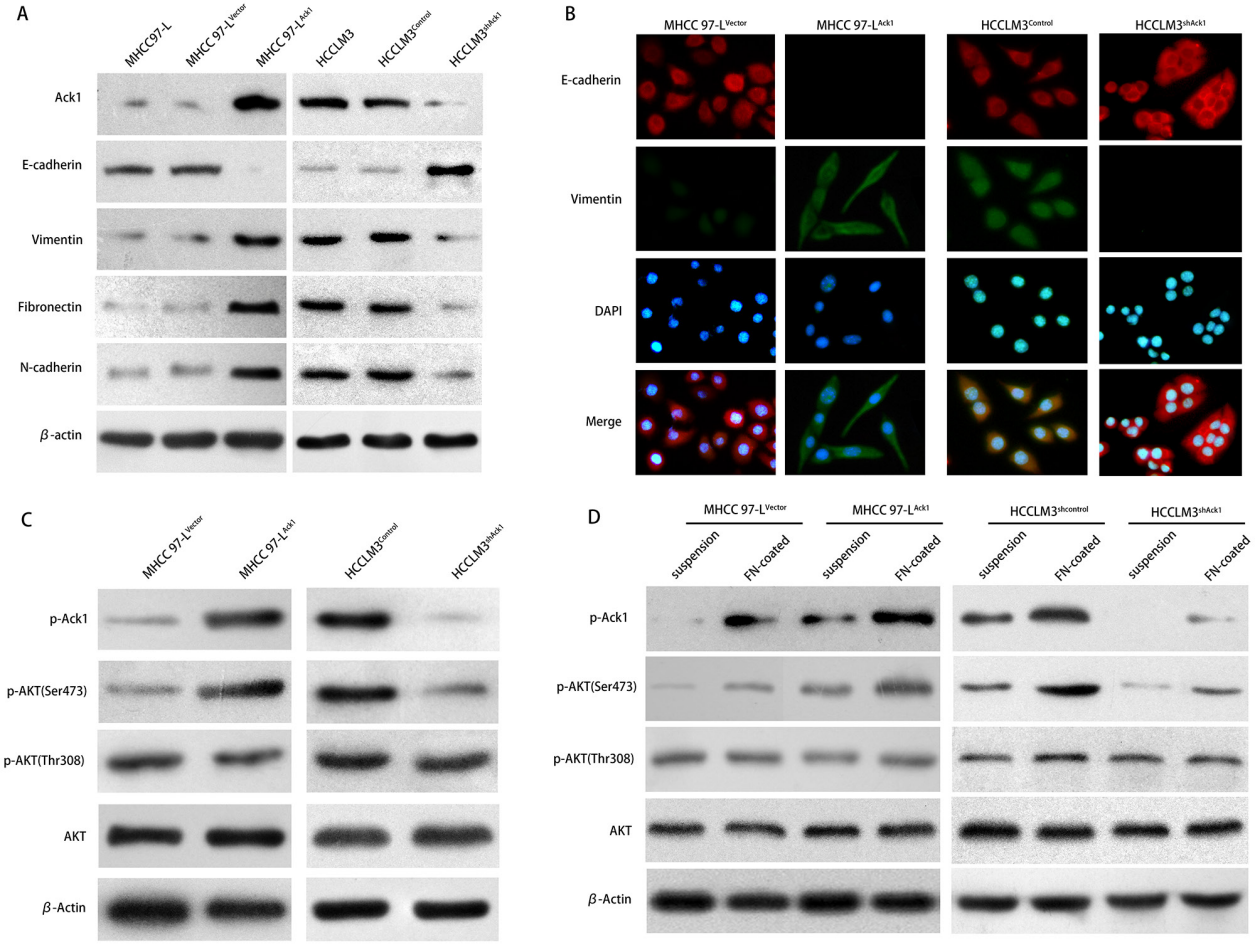
Ack1 enhances HCC cells metastasis via EMT by mediating phosphorylation on Ser473 of AKT **A.** Western blot analyzed Ack1 and EMT markers expression in HCC cells with Ack1 overexpression or knockdown, and their corresponding parental, control cells. **B.** Immunofluorescent (IF) showed the relative expression of E-cadherin (red), vimentin (green), and 4′, 6-diamidino-2-phenylindole (DAPI; blue) in HCC cells with Ack1 overexpression or knockdown, their corresponding control cells. **C.** Expression of Tyr-phosphorylation of Ack1 and key proteins in AKT signaling and its phosphorylation in MHCC97-L and HCCLM3 cell lines were detected by western blot. **D.** Cell adhesion assays showed overexpression of Ack1 increased Ser473-phosphorylation of AKT upon fibronectin stimulation in MHCC97-L cells, while knockdown of Ack1 decreased Ser473-phosphorylation of AKT upon fibronectin stimulation in HCCLM3 cells. Cell adhesion assays were determined as described in Materials and Methods.

Ack1 overexpression could induce the reorganization of actin cytoskeleton and modulate HCC cells into a mesenchymal-like morphology (Supplementary Figure S3C), while down-regulation of Ack1 changed the cell appearance to more cobble-stone morphology (Supplementary Figure S3D), indicating that Ack1 was involved in a cellular transition termed epithelial-mesenchymal transition (EMT). [22] To confirm whether Ack1 promotes HCC metastasis by facilitating EMT, we examined the expression levels of EMT markers cells or knockdown of Ack1 in HCCLM3 cells. Compared with control cells (MHCC97-L^Vector^ cells), western blot showed that Ack1-overexpression cells (MHCC97-L^Ack1^ cells) exhibited a large decrease in epithelial-marker E-cadherin and increase in mesenchymal markers vimentin, fibronectin and N-cadherin (Figure 5A). However, western blot showed that knockdown of Ack1 increased epithelial-marker E-cadherin and decreased mesenchymal markers vimentin, fibronectin and N-cadherin expression in HCCLM3 cells (Figure 5A). Immunofluorescent (IF) assays also revealed that overexpression of Ack1 in MHCC97-L cells increased the expression of vimentin, but decreased the expression of E-cadherin (Figure 5B). Conversely, knockdown of Ack1 in HCCLM3 cells increased the expression of E-cadherin, but decreased the expression of vimentin (Figure 5B). These results supported that Ack1 was associated with EMT in HCC cells.

To explore essential signaling pathways that are capable of conferring metastatic phenotypes to HCC cells, we determined whether Ack1 affected signaling pathways that have clearly established roles in promoting HCC metastasis via EMT. It has been shown that Ack1 is activated by overexpression of the Ack1 gene and cell adhesion. [23] We observed that Ack1 overexpression cells MHCC97-L^Ack1^ yielded much more Tyr-phosphorylation of Ack1 than control cells MHCC97-L^Vector^, while Ack1-knockdown cells HCCLM3^shAck1^ yielded much less Tyr-phosphorylation of Ack1 than control cells HCCLM3^shcontrol^ (Figure 5C). It was reported that the kinase activation of Ack1 could activate AKT pathway in other disease, [23, 24] which is important for epithelial-mesenchymal transition (EMT). [21] Thus we investigated the effect of Ack1 on Thr308- and Ser473-phosphorylation of AKT. Western blot revealed that Ack1 overexpression significantly induced phosphorylation on Ser473 of AKT, but not on Thr308 of AKT (Figure 5C). To substantiate AKT activation mediated by Ack1, we tested the results by cell adhesion assays (Figure 5D). HCC cells MHCC97-L^Ack1^, MHCC97-L^Vector^, HCCLM3^shcontrol^ and HCCLM3^shAck1^ cells were plated onto fibronectin plates. As expected, western blot showed Tyr-phosphorylation of Ack1 was significantly enhanced upon plating the cells onto fibronectin (Figure 5D). In MHCC97-L cells, cell adhesion assays also showed Ser473-phosphorylation of AKT was enhanced upon plating the cells onto fibronectin, but Ser473-phosphorylation of AKT was greater in MHCC97-L^Ack1^ cells than in MHCC97-L^Vector^ cells (Figure 5D). Similarly, in HCCLM3 cells, cell adhesion assays showed Ser473-phosphorylation of AKT was enhanced upon plating the cells onto fibronectin, but Ser473-phosphorylation of AKT was greater in HCCLM3^shcontrol^ cells than in HCCLM3^shAck1^ cells (Figure 5D). However, Thr308-phosphorylation of AKT was yet not affected in MHCC97-L cells or HCCLM3 cells (Figure 5D). To assess the role of Ack1 in Ser473-phosphorylation of AKT, HCC cells were either untreated or pretreated with AIM-100, a Ack1-specific inhibitor, in cell adhesion assays (Supplementary Figure S5A and S5B). This assays further validated that the kinase activation of Ack1 could activated AKT pathway by inducing Ser473-phosphorylation of AKT, but not Thr308-phosphorylation of AKT in HCC cells. Taken together, these data suggest that Ack1 can promote EMT by mediating activation of AKT signaling pathway in HCC.

## DISCUSSION

In this study, we identified overexpression of Ack1 related to HCC metastasis and predictive prognosis after liver resection according to REMARK guidelines for reporting prognostic biomarkers in cancer. [19] We demonstrated that Ack1 was frequently overexpressed in human HCC tumors compared with ANLTs. IHC also showed the expression level of Ack1 was progressively increased from ANLTs, PLs to IMLs. Interestingly, among three subtypes including SHCC, SLHCC and NHCC, Ack1 expression level was the lowest in SLHCC with the best behavior of oncological biology according to our clinical outcome. [2, 4] Further analysis showed high Ack1 expression was clearly associated with tumor differentiation, vascular invasion, pathologic satellite nodules, and the TNM stage of tumors. These data were consistent with previous reports that Ack1 was implicated in cancer progression. [25] However, high Ack1 expression correlated with TNM stage but not BCLC stage, which may be due to the fact that these two staging systems use different variables related to tumor status, liver function and health performance status. [26, 27] Anyhow, tumors in advanced TNM stage possessed high Ack1 levels, and this result consolidated the view that Ack1 overexpression related to cancer progression. Taken together, these findings indicate that Ack1 functions as an oncogene promoting HCC metastasis.

HCC is a highly heterogeneous disease that patients with the same pathologic features, TNM stage and treatment strategy (such as curative resection) could have different clinical outcomes. [28] Therefore, it is essential to find powerful markers to predict the tumor recurrence after curative liver resection. In this study, we aimed to explore whether Ack1 expression level was related to the prognosis of HCC according to REMARK guidelines. [19] We proved that high Ack1 expression was an independent and significant factor for DFS and OS for HCC, specially for early recurrence (within 2 years). Early recurrence mainly results from dissemination of metastatic HCC cells involved in HCC cell biology. [29] The predictive potential of Ack1 was found in early recurrence, implying HCC cells with high Ack1 expression are predisposed to become metastatic. Indeed, our results showed that Ack1 could enhance HCC cells motility, invasion, and epithelial-mesenchymal transition. The results also explained that patients with high Ack1 expression have markedly poor prognosis, and the varying Ack1 expression is a reason for the different clinical outcomes between SLHCC and NHCC. [2, 4] In addition, we validated the prognosis significance in internal cohort of HCC demonstrating its powerful performance in predicting prognosis, and further validated its prognostic accuracy in external cohort of HCC. These results support Ack1 could be a useful predictive marker for prognosis in addition to metastasis in HCC.

We demonstrated that Ack1 played its role in promoting invasion and metastasis of HCC. Our study on HCC cell lines showed that Ack1 was overexpressed in HCC cell lines and the highest expression occurred in HCCLM3 cell line with the highest metastasis potential. [20, 21] Overexpression of Ack1 in MHCC97-L cells markedly promoted cell migration and invasion, while knockdown of Ack1 in HCCLM3 cells significantly decreased cell migration and invasion. Importantly, an animal model confirmed that Ack1 facilitated tumor formation and metastasis *in vivo*. These further suggest that Ack1 promotes the invasion and metastasis of HCC.

EMT is considered as a critical mechanism contributing to metastasis of cancer. [21, 30, 31] Previous studies have shown that Ack1 could modulate EMT. [32] In this study, we confirmed that Ack1 enhanced HCC metastasis via EMT, as EMT markers such as E-cadherin, vimentin, fibronectin, and N-cadherin were affected by Ack1 ectopic expression or knockdown. E-cadherin is the key components of the adherens junctions of cell membrane mediating cell-cell adhesion and cytoskeleton. [33] Our data showed that overexpression of Ack1 up-regulated E-cadherin expression, while Ack1 knockdown down-regulated E-cadherin expression. We also uncovered Ack1 exerted function via the oncogene AKT in HCC. AKT is frequently activated in many cancer types and has emerged as a major hallmark of tumor metastasis. [21, 34, 35] Its activation may be in the different activation fashions, and the amino acid residues of different phosphorylation sites. [36, 37] Recently, we also reported that phosphorylation of Ser473 in the AKT is an important signal for HCC metastasis. [21] In this study, Ack1-mediated phosphorylation of Ser473 in the AKT is an another evidence for our previous study. It has been shown that Ack1 is activated by cell adhesion and is a critical transducer of multiple receptor signals, including EGFR, PDGFR (platelet-derived growth factor receptor), insulin receptor. [31, 38, 39]. Activation of Ack1 directly phosphorylates AKT at an evolutionarily conserved tyrosine 176 in the kinase domain, which localizes to the plasma membrane and promotes Ser473-phosphorylation leading to AKT activation. [24] Cell adhesion assays further tested the view and suggested that Ack1 could mediate activation of AKT by phosphorylation on Ser473. However, phosphorylation of Thr308 in the AKT was not affected by Ack1, but its high phosphorylation was detected in HCC cells. This may be that HCC cells often have a loss of PTEN, [40] leading to constitutively activated AKT, [41] which could already lead to discrepancies in phosphorylation of the amino acid residues of AKT in HCC. All together, these results indicate that Ack1 serves as an EMT promoter through activating AKT signaling pathway.

In conclusion, our study, according to REMARK guidelines, have identified the HCC prognosis of Ack1 in 2 independent cohorts using different techniques and further validated its accuracy of a prognostic biomarker in external cohort of resected HCC patients. We found that Ack1 was overexpressed in HCC and Ack1 promoted HCC metastasis through EMT by activating AKT signaling pathway. Also, Ack1 may be a potential prognostic biomarker for the patients with HCC after liver resection, supporting the pursuit of clinical significance of Ack1.

## MATERIALS AND METHODS

### HCC patients and tissue specimens

Three independent cohorts of HCC subjects were enrolled in this study (Supplementary Figure S1). All patients without any preoperative treatment were histopathologically confirmed as HCC according to World Health Organization criteria and pathologically staged based on the Barcelona clinic liver cancer (BCLC) staging scores and tumor node metastasis (TNM) classification (7th Edition) of the International Union Against Cancer. [26, 27] In training cohort (*n* = 76), fresh matched specimens of HCCs and ANLTs were randomly collected from HCC patients undergoing hepatic resection between June 2006 and April 2008 in the Department of Surgery, Xiangya Hospital, Central South University (CSU), China. In internal validation cohort (*n* = 141), paraffin-embedded specimens were randomly collected from HCC patients undergoing curative resection in Xiangya Hospital of CSU from October 2001 to April 2013. In external validations cohort (*n* = 70), paraffin embedded specimens were randomly collected at another research center–The Affiliated Cancer Hospital of Xiangya School of Medicine, CSU from January 2003 to December 2013. Detail demographics and clinicopathological characteristics were described in the Supplementary Table 1. Prior informed consent was obtained and the study protocol was approved by the Ethics Committee of Xiangya School of Medicine, CSU.

### Follow-up and prognostic studies

All patients of training and internal validation cohort were regularly followed-up by the same experienced surgical team in our liver surgery center, with surveillance for the recurrence and metastasis by clinical examination, serial monitoring of alpha-fetoprotein levels and ultrasonography or CT scan or MRI at a 3–4 month interval. The median follow up was 46.5 months (range from 3–120 months). For external validation cohort, followed-up data were collected by the experienced surgical team in this center. All of us were blinded to survival data before analysis. For the patients suspected recurrence and/or metastasis, imaging examinations including high-resolution contrast-enhanced CT and/or magnetic resonance imaging (MRI) were performed to validate recurrent or metastatic lesions. Overall survival (OS) was defined as the time interval between HCC resection and death or the last observation. Patients alive at the end of follow up were censored. Disease-free survival (DFS) is calculated from the HCC resection to the first radiological evidence of recurrence. Patients that died with no evidence of recurrence were censored.

### Cell lines and cell culture

HCCLM3 and MHCC97-L cell lines were gifted by Liver Cancer Institute of Fudan University, Shanghai, China. HepG2, Huh7 and L02 cell lines were purchased from American Type Culture Collection (ATCC, Rockville, MA). Cell lines were cultured in high glucose Dulbecco's modified Eagle media (DMEM) (GIBCO BRL, Gaithersburg, MD) supplemented with 10% fetal bovine serum (HyClone, Logan, UT) and maintained in 5% CO2 at 37°C.

### Quantitative real-time pcr

Quantitative real-time (qRT PCR) was performed as we previously described. [21] For qRT-PCR, primers were as follows: Ack1-forward, 5′- TGCCACAAAGTGCTGGAGATG-3′; Ack1-reverse, 5′- ACGGACAGGCTCAGGTGATTC-3′; GAPDH- forward, 5′-GCACCGTCAAGGCTGAGAAC-3′; GAPDH-reverse, 5′- TGGTGAAGACGCCAGTGGA-3′. The PCR cycling parameters were as follows: 50 cycles at 95°C for 5s, 60°C for 20s. Relative mRNA expression levels were calculated by the 2^−ΔCt^ method based on the threshold cycle (Ct) values and were normalized to the internal control of GAPDH.

### Western blot

Total protein was extracted and separated by SDS-PAGE and then transferred onto PVDF membrane (Millipore, Bedford, MA). The blotted membranes were incubated with the primary antibodies and then HRP-conjugated goat anti mouse secondary antibody (1:2,000; KPL, Gaithersburg, MD) in order. Band was detected with enhanced chemiluminescence regents (Thermo Scientific, Rockford, IL). Beta-actin protein was also determined by using the specific antibody (Sigma, St Louis, MO) as a loading control. Protein expression were quantified by BandScan software (BioRad, Hercules, CA) and defined as the ratio of target protein relative to Beta-actin. Antibodies for E-cadherin, vimentin, fibronectin, N-cadherin, p-Ack1(Tyr284), Ack1, p-AKT(Thr308), p-AKT(Ser473) and corresponding secondary antibodies were purchased from Santa Cruz Biotechnology (Santa Cruz Biotechnology, Santa Cruz, CA).

### Immunohistochemistry

Paraffin-embedded tissues were sectioned and microwave-pretreated in EDTA buffer (1 mM, pH 8.0) for 10 minutes for antigen retrieval. Immunostaining for Ack1 (1:200) (Santa Cruz Biotechnology) was done with the Streptavidin-Peroxidase system (Zhongshan Goldenbridge Biotechnology, Shanghai, China). The expression levels of Ack1 were scored semiquantitatively based on staining intensity and percentage of positive cells using the immunostaining score as described elsewhere. [42] Briefly, immunostaining score (IS) = SI(staining intensity) × PP (percentage of positive cells). SI was classified as: 0, negative; 1, weak; 2, moderate; 3, strong. PP was defined as: 0, 0% positive cells; 1, 0–25% positive cells; 2, 25–50% positive cells; 3, 50–75% positive cells, and 4, 75–100% positive cells. For categorization of the continuous Ack1 values into low and high, we chose a commonly used cutoff point for the analysis (range 0–12, cut point ≤ 3 versus > 3). Scoring was performed separately by two experienced pathologist.

### Expression vectors construction and transfection

For Ack1 overexpression, pCMV-Tag2B was a generous gift from Dr. Yuan. Ack1-expression plasmid pCMV-Tag2B-Ack1 was synthesize and kindly provided by Amgen Company, USA. Exponentially growing cells MHCC97-L (about 60–80% confluent) transfected with pCMV-Tag2B-Ack1 or empty vectors using FuGENE 6 (Invitrogen, Carlsbad, CA) and selected with G418 (300 μg/ml). For Ack1 knockdown, vector GV112 plasmids carrying shRNA for Ack1 or scrambled shRNA were purchased from GeneChem corporation (Shanghai, China). Three putative candidate Ack1 shRNA sequences and one control sequence are included below: Ack1-shRNA-Seq1: Sense: 5′-CCGGGTCGTGGATGAGTAAGGTGTTCTCG AGAACACCTTACTCATCCACGACTTTTTG-3′. Ack1- shRNA-Seq2: Sense: 5′-CCGGTGCTTCCTCTTCC ACCCAATTCTCGAGAATTGGGTGGAAGAGGAAGC ATTTTTG-3′. Ack1-shRNA-Seq3: Sense: 5′-CCGGCA ACTTCTCCACCAACAACAGCTCGAGCTGT TGTTG GTGGAGAAGTT GTTTTTG-3′. Control shRNA sequence: Sense: 5′-CCGGCATTCTCCGAACGTG TCACGTCTCGAGACGTGACACGTTCGGAGAATGT TTTTG-3′. Exponentially growing cells HCCLM3 (about 60–80% confluent) were transfected with either shRNA plasmids for Ack1 or for control using FuGENE 6 (Invitrogen) and selected with 3 μg/ml puromycin (Sigma, St. Louis, MO).

### Immunofluorescence analysis

Stably transfected cells were seeded in the 6-well Culture plate (Corning Costar Corp, Corning, NY) to prepare for performed cell immunofluorescence (IF) and incubated with primary antibodies then incubated with fluorescence labeled secondary antibody. The slides were imaged using a microscope or inverted fluorescence microscope TE-2000S (Nikon, Tokyo, Japan). Primary antibodies for E-cadherin, vimentin were purchased from Santa Cruz Biotechnology (Santa Cruz, CA). Rhodamine-conjugated phalloidin, DAPI and fluorescence labeled secondary antibody were obtained from Beyotime Institute of Biotechnology (Shanghai, China).

### Cell proliferation and colony formation

HCC cell lines proliferation and colony formation were performed and analyzed as described previously. [21] For cell proliferation assay, cell numbers were counted by using cell counter and then compared. For colony formation assays, single-cell suspension containing 500 cells was added to each well of a 6-well plate (Corning Costar Corp) and incubated in 5% CO_2_ at 37°C for 2 weeks. Then, fixed with methanol and stained with crystal violet (Beyotime, Shanghai, China). Only positive colonies (diameter > 40 um) in the dishes were counted and compared. [43] These experiments were performed in triplicate.

### Wound-healing assays

Wound-healing assays were performed and analyzed as described elsewhere. [44] In brief, Cells were seeded onto 35 mm dishes (Corning Costar Corp) coated with fibronectin. After reached 100% confluence, cells were pre-incubated with mitomycin (Sigma, St. Louis, MO, 10 μg/ml) to inhibit cell proliferation for 1 h at 37°C. Wound-healing assays were performed with a sterile pipette tip to make a scratch through the confluent monolayer cell. Medium was changed and cells were cultured for 24 hours. The percent of wound closure was calculated for five randomly chosen fields.

### Transwell assays

Transwell assays were performed and analyzed as described. [42] Briefly, 1 × 10^5^ cells in DMEM medium containing 0.1% bovine serum albumin were placed into the upper chamber of the insert with matrigel (BD Biosciences, Franklin Lakes, NJ). After 24 hours of incubation at 37°C, we removed the cells remaining in the upper chamber. After fixing with 20% methanol and staining with a solution containing 0.1% crystal violet (Beyotime Institute of Biotechnology, Beijing, China), the number of cells adhering to the lower membrane of the inserts was counted. For each experiment group, the invasion assay was performed in triplicate, and five randomly fields of each replicate were chosen for analysis.

### Cell adhesion assay

The cell adhesion assay was used to determine the activation of Ack1 performed as described elsewhere. [23] In brief, non-coated common culture plates were incubated with fibronectin (10 μg/ml) or PBS (the solvent for fibronectin) for 12 h at 4°C followed by blocking with 2% BSA for 2 h at 37°C. The plates incubated with PBS (used for cells in suspension) were set as control, and ones incubated with fibronectin (used for cells in adherency) were set as experiment group. The cells, which were transfected with Ack1 or its vector, were detached from the plates by incubation in 2% EGTA and washed three times with PBS. The suspended cells were incubated on the control or the fibronectin-coated plates for 30 min. For Ack1 inhibitor assays, HCC cells (1 × 10^5^/ml) were untreated or treated with 4 μM AIM-100 (Medchemexpress, Monmouth, NJ) for 24 h, [45] then following incubated on the control or the fibronectin-coated plates for 30 min. Both suspended and adhered cells were harvested and lysed. The tyrosine phosphorylation of ACK1 was detected by western-blot with an anti-p-Ack1 (Tyr284) antibody (Santa Cruz Biotechnology).

### Tumor formation and metastasis assays *in vivo*

A metastatic hepatocellular carcinoma model in nude mice (3–4 weeks of age, male, BALB/c) was established according to the existed protocol. [21] Briefly, approximately 5 × 10^6^ cells within 0.2 mL culture medium without FBS were injected subcutaneously into the right flank of the mice. After 3 weeks, subcutaneous tumors were obtained and divided into commensurate fragments of approximately 1 mm^3^. One fragment was implanted into the left liver lobe of another mouse (seven mice for each group). After 35days, the animals were sacrificed by cervical dislocation and autopsied. The length (L) and width (W) of tumors were measured at autopsy and volume (V) was calculated as follows: *V* = 1/2(L × W^2^). [46] The nodules that were distant from the *in situ* tumors were defined as intrahepatic metastasis. Lung tissue of each mouse was harvested, fixed, embedded, sectioned serially, stained with hematoxylin and eosin (H&E), and observed under a microscope. Mice whose metastatic HCC nodules were found on any slide of lung sections were labeled as pulmonary metastasis presented. The number and grade of metastatic intrahepatic and lung nodules were detected using microscopy. The grade of metastases were classified based on the number of tumor cells present at the maximal section in each metastatic lesion (grade I, ≤ 20; grade II, 20–50; grade III, 50–100; and grade IV, ≥ 100 tumor cells). [22]

### Statistical analysis

All analyses were performed by SPSS18.0 software (SPSS, Chicago, IL). Categorical data was analyzed by χ^2^ test. Continuous data was presented as mean ± S.D. and analyzed by *t* tests or Mann-Whitney *U*-test when variances are unequal. Survival curves were plotted using the Kaplan-Meier method and analyzed using the log-rank test. The univariate and multivariate Cox proportional hazards regression model was used to identify independent risk factors for overall survival (OS) and disease-free survival (DFS). All tests were two tailed and *P* < 0.05 was considered as statistically significant.

## SUPPLEMENTARY FIGURES AND TABLES



## References

[1] Torre LA, Bray F, Siegel RL, Ferlay J, Lortet-Tieulent J, Jemal A (2015). Global cancer statistics, 2012. CA Cancer J Clin.

[2] Yang LY, Chang RM, Lau WY, Ou DP, Wu W, Zeng ZJ (2014). Mesohepatectomy for centrally located large hepatocellular carcinoma: Indications, techniques, and outcomes. SURGERY.

[3] Liao W, Huang G, Liao Y, Yang J, Chen Q, Xiao S, Jin J, He S, Wang C (2014). High KIF18A expression correlates with unfavorable prognosis in primary hepatocellular carcinoma. ONCOTARGET.

[4] Yang LY, Fang F, Ou DP, Wu W, Zeng ZJ, Wu F (2009). Solitary large hepatocellular carcinoma: a specific subtype of hepatocellular carcinoma with good outcome after hepatic resection. ANN SURG.

[5] Shim JH, Jun MJ, Han S, Lee YJ, Lee SG, Kim KM, Lim YS, Lee HC (2015). Prognostic nomograms for prediction of recurrence and survival after curative liver resection for hepatocellular carcinoma. ANN SURG.

[6] Yang LY, Wang W, Peng JX, Yang JQ, Huang GW (2004). Differentially expressed genes between solitary large hepatocellular carcinoma and nodular hepatocellular carcinoma. World J Gastroenterol.

[7] Wang W, Peng JX, Yang JQ, Yang LY (2009). Identification of gene expression profiling in hepatocellular carcinoma using cDNA microarrays. Dig Dis Sci.

[8] Wang W, Wu F, Fang F, Tao Y, Yang L (2008). Inhibition of invasion and metastasis of hepatocellular carcinoma cells via targeting RhoC *in vitro* and *in vivo*. CLIN CANCER RES.

[9] Wang W, Wu F, Fang F, Tao Y, Yang L (2008). RhoC is essential for angiogenesis induced by hepatocellular carcinoma cells via regulation of endothelial cell organization. CANCER SCI.

[10] Wang W, Yang LY, Yang ZL, Peng JX, Yang JQ (2007). Elevated expression of autocrine motility factor receptor correlates with overexpression of RhoC and indicates poor prognosis in hepatocellular carcinoma. Dig Dis Sci.

[11] Mahajan K, Mahajan NP (2015). ACK1/TNK2 tyrosine kinase: molecular signaling and evolving role in cancers. ONCOGENE.

[12] Manser E, Leung T, Salihuddin H, Tan L, Lim L (1993). A non-receptor tyrosine kinase that inhibits the GTPase activity of p21cdc42. NATURE.

[13] Mahajan NP, Liu Y, Majumder S, Warren MR, Parker CE, Mohler JL, Earp HS, Whang YE (2007). Activated Cdc42-associated kinase Ack1 promotes prostate cancer progression via androgen receptor tyrosine phosphorylation. Proc Natl Acad Sci U S A.

[14] Kiyose S, Nagura K, Tao H, Igarashi H, Yamada H, Goto M, Maeda M, Kurabe N, Suzuki M, Tsuboi M, Kahyo T, Shinmura K, Hattori N, Sugimura H (2012). Detection of kinase amplifications in gastric cancer archives using fluorescence *in situ* hybridization. PATHOL INT.

[15] Mahajan K, Coppola D, Chen YA, Zhu W, Lawrence HR, Lawrence NJ, Mahajan NP (2012). Ack1 tyrosine kinase activation correlates with pancreatic cancer progression. AM J PATHOL.

[16] Modzelewska K, Newman LP, Desai R, Keely PJ (2006). Ack1 mediates Cdc42-dependent cell migration and signaling to p130Cas. J BIOL CHEM.

[17] van der Horst EH, Degenhardt YY, Strelow A, Slavin A, Chinn L, Orf J, Rong M, Li S, See LH, Nguyen KQ, Hoey T, Wesche H, Powers S (2005). Metastatic properties and genomic amplification of the tyrosine kinase gene ACK1. Proc Natl Acad Sci U S A.

[18] Eisenmann KM, McCarthy JB, Simpson MA, Keely PJ, Guan JL, Tachibana K, Lim L, Manser E, Furcht LT, Iida J (1999). Melanoma chondroitin sulphate proteoglycan regulates cell spreading through Cdc42, Ack- and p30cas. NAT CELL BIOL.

[19] McShane LM, Altman DG, Sauerbrei W, Taube SE, Gion M, Clark GM (2005). Reporting recommendations for tumor marker prognostic studies (REMARK). J Natl Cancer Inst.

[20] Tang ZY, Ye SL, Liu YK, Qin LX, Sun HC, Ye QH, Wang L, Zhou J, Qiu SJ, Li Y, Ji XN, Liu H, Xia JL, Wu ZQ, Fan J, Ma ZC (2004). A decade's studies on metastasis of hepatocellular carcinoma. J Cancer Res Clin Oncol.

[21] Chang RM, Yang H, Fang F, Xu JF, Yang LY (2014). MicroRNA-331-3p promotes proliferation and metastasis of hepatocellular carcinoma by targeting PH domain and leucine-rich repeat protein phosphatase. HEPATOLOGY.

[22] Zhu K, Dai Z, Pan Q, Wang Z, Yang GH, Yu L, Ding ZB, Shi GM, Ke AW, Yang XR, Tao ZH, Zhao YM, Qin Y, Zeng HY, Tang ZY, Fan J (2011). Metadherin promotes hepatocellular carcinoma metastasis through induction of epithelial-mesenchymal transition. CLIN CANCER RES.

[23] Lin Q, Wang J, Childress C, Yang W (2012). The activation mechanism of ACK1 (activated Cdc42-associated tyrosine kinase 1). BIOCHEM J.

[24] Mahajan K, Coppola D, Challa S, Fang B, Chen YA, Zhu W, Lopez AS, Koomen J, Engelman RW, Rivera C, Muraoka-Cook RS, Cheng JQ, Schonbrunn E, Sebti SM, Earp HS, Mahajan NP (2010). Ack1 mediated AKT/PKB tyrosine 176 phosphorylation regulates its activation. PLOS ONE.

[25] Mahajan K, Challa S, Coppola D, Lawrence H, Luo Y, Gevariya H, Zhu W, Chen YA, Lawrence NJ, Mahajan NP (2010). Effect of Ack1 tyrosine kinase inhibitor on ligand-independent androgen receptor activity. PROSTATE.

[26] European Association for the Study of the Liver, European Organisation for Research and Treatment of Cancer (2012). EASL-EORTC clinical practice guidelines: management of hepatocellular carcinoma. J HEPATOL.

[27] Edge SB, Byrd DR, Compton CC, Fritz AG, Greene FL, Trotti A (2010). AJCC Cancer Staging Handbook.

[28] Faber W, Sharafi S, Stockmann M, Denecke T, Sinn B, Puhl G, Bahra M, Malinowski MB, Neuhaus P, Seehofer D (2013). Long-term results of liver resection for hepatocellular carcinoma in noncirrhotic liver. SURGERY.

[29] Portolani N, Coniglio A, Ghidoni S, Giovanelli M, Benetti A, Tiberio GA, Giulini SM (2006). Early and late recurrence after liver resection for hepatocellular carcinoma: prognostic and therapeutic implications. ANN SURG.

[30] Jou J, Diehl AM (2010). Epithelial-mesenchymal transitions and hepatocarcinogenesis. J CLIN INVEST.

[31] Mitra A, Mishra L, Li S (2015). EMT, CTCs and CSCs in tumor relapse and drug-resistance. ONCOTARGET.

[32] Chua BT, Lim SJ, Tham SC, Poh WJ, Ullrich A (2010). Somatic mutation in the ACK1 ubiquitin association domain enhances oncogenic signaling through EGFR regulation in renal cancer derived cells. MOL ONCOL.

[33] Lu M, Marsters S, Ye X, Luis E, Gonzalez L, Ashkenazi A (2014). E-cadherin couples death receptors to the cytoskeleton to regulate apoptosis. MOL CELL.

[34] Manning BD, Cantley LC (2007). AKT/PKB signaling: navigating downstream. CELL.

[35] Vivanco I, Sawyers CL (2002). The phosphatidylinositol 3-Kinase AKT pathway in human cancer. NAT REV CANCER.

[36] Moore NF, Azarova AM, Bhatnagar N, Ross KN, Drake LE, Frumm S, Liu QS, Christie AL, Sanda T, Chesler L, Kung AL, Gray NS, Stegmaier K, George RE (2014). Molecular rationale for the use of PI3K/AKT/mTOR pathway inhibitors in combination with crizotinib in ALK-mutated neuroblastoma. ONCOTARGET.

[37] Sarbassov DD, Guertin DA, Ali SM, Sabatini DM (2005). Phosphorylation and regulation of Akt/PKB by the rictor-mTOR complex. SCIENCE.

[38] Galisteo ML, Yang Y, Urena J, Schlessinger J (2006). Activation of the nonreceptor protein tyrosine kinase Ack by multiple extracellular stimuli. Proc Natl Acad Sci U S A.

[39] Mahajan NP, Liu Y, Majumder S, Warren MR, Parker CE, Mohler JL, Earp HS, Whang YE (2007). Activated Cdc42-associated kinase Ack1 promotes prostate cancer progression via androgen receptor tyrosine phosphorylation. Proc Natl Acad Sci U S A.

[40] Zhang Y, Zheng L, Ding Y, Li Q, Wang R, Liu T, Sun Q, Yang H, Peng S, Wang W, Chen L (2015). MiR-20a Induces Cell Radioresistance by Activating the PTEN/PI3K/Akt Signaling Pathway in Hepatocellular Carcinoma. Int J Radiat Oncol Biol Phys.

[41] Hales EC, Orr SM, Larson GA, Taub JW, Matherly LH (2013). Notch1 receptor regulates AKT protein activation loop (Thr308) dephosphorylation through modulation of the PP2A phosphatase in phosphatase and tensin homolog (PTEN)-null T-cell acute lymphoblastic leukemia cells. J BIOL CHEM.

[42] Su S, Liu Q, Chen J, Chen J, Chen F, He C, Huang D, Wu W, Lin L, Huang W, Zhang J, Cui X, Zheng F, Li H, Yao H, Su F (2014). A positive feedback loop between mesenchymal-like cancer cells and macrophages is essential to breast cancer metastasis. CANCER CELL.

[43] Garcia-Echeverria C, Pearson MA, Marti A, Meyer T, Mestan J, Zimmermann J, Gao J, Brueggen J, Capraro HG, Cozens R, Evans DB, Fabbro D, Furet P, Porta DG, Liebetanz J, Martiny-Baron G (2004). *In vivo* antitumor activity of NVP-AEW41-A novel, potent, and selective inhibitor of the IGF-IR kinase. CANCER CELL.

[44] Pullar CE, Chen J, Isseroff RR (2003). PP2A activation by beta2-adrenergic receptor agonists: novel regulatory mechanism of keratinocyte migration. J BIOL CHEM.

[45] Mahajan K, Challa S, Coppola D, Lawrence H, Luo Y, Gevariya H, Zhu W, Chen YA, Lawrence NJ, Mahajan NP (2010). Effect of Ack1 tyrosine kinase inhibitor on ligand-independent androgen receptor activity. The Prostate.

[46] Zhang JF, He ML, Fu WM, Wang H, Chen LZ, Zhu X, Chen Y, Xie D, Lai P, Chen G, Lu G, Lin MC, Kung HF (2011). Primate-specific microRNA-637 inhibits tumorigenesis in hepatocellular carcinoma by disrupting signal transducer and activator of transcription 3 signaling. HEPATOLOGY.

