# Supplementary material for: Ack1 overexpression promotes metastasis and indicates poor prognosis of hepatocellular carcinoma
Source: Oncotarget. 2015 Oct 20;6(38):40622–41. doi: 10.18632/oncotarget.5872 (PMC4747357; doi:10.18632/oncotarget.5872)
Supplement: Supplementary file 1 [file oncotarget-06-40622-s001.pdf]

SUPPLEMENTARY FIGURES AND TABLES

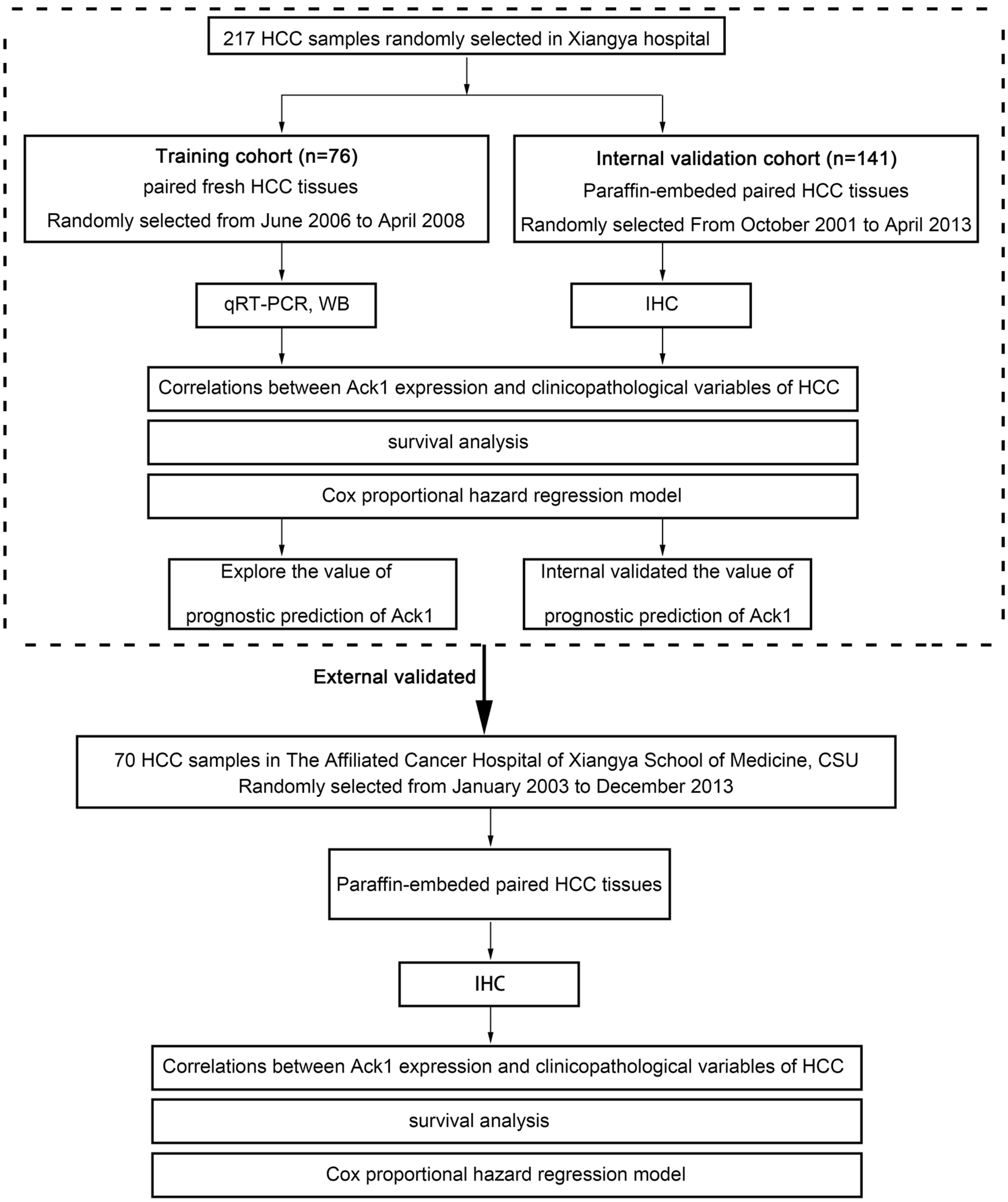

Supplementary Figure S1: A flow chart of study profile in three independent cohorts of HCC patients. qRT-PCR, WB, and IHC respectively represents quantitative RT-PCR, western blot, and immunohistochemistry.

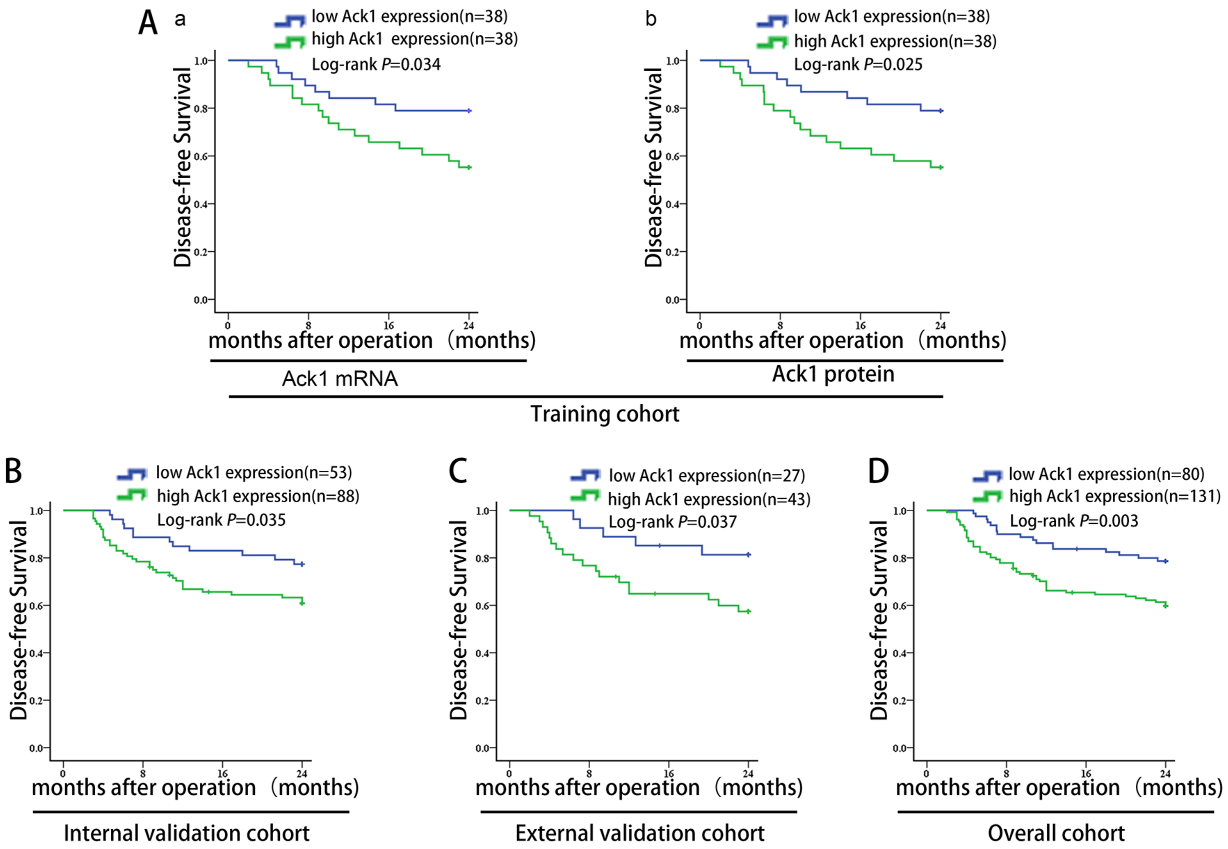

Supplementary Figure S2: Kaplan-Meier analysis of the relationship between Ack1 expression and early recurrence (≤ 24 months) of HCC patients in training cohort. A. internal validation cohort **B.** external validation cohort **C.** and overall cohort **D.**

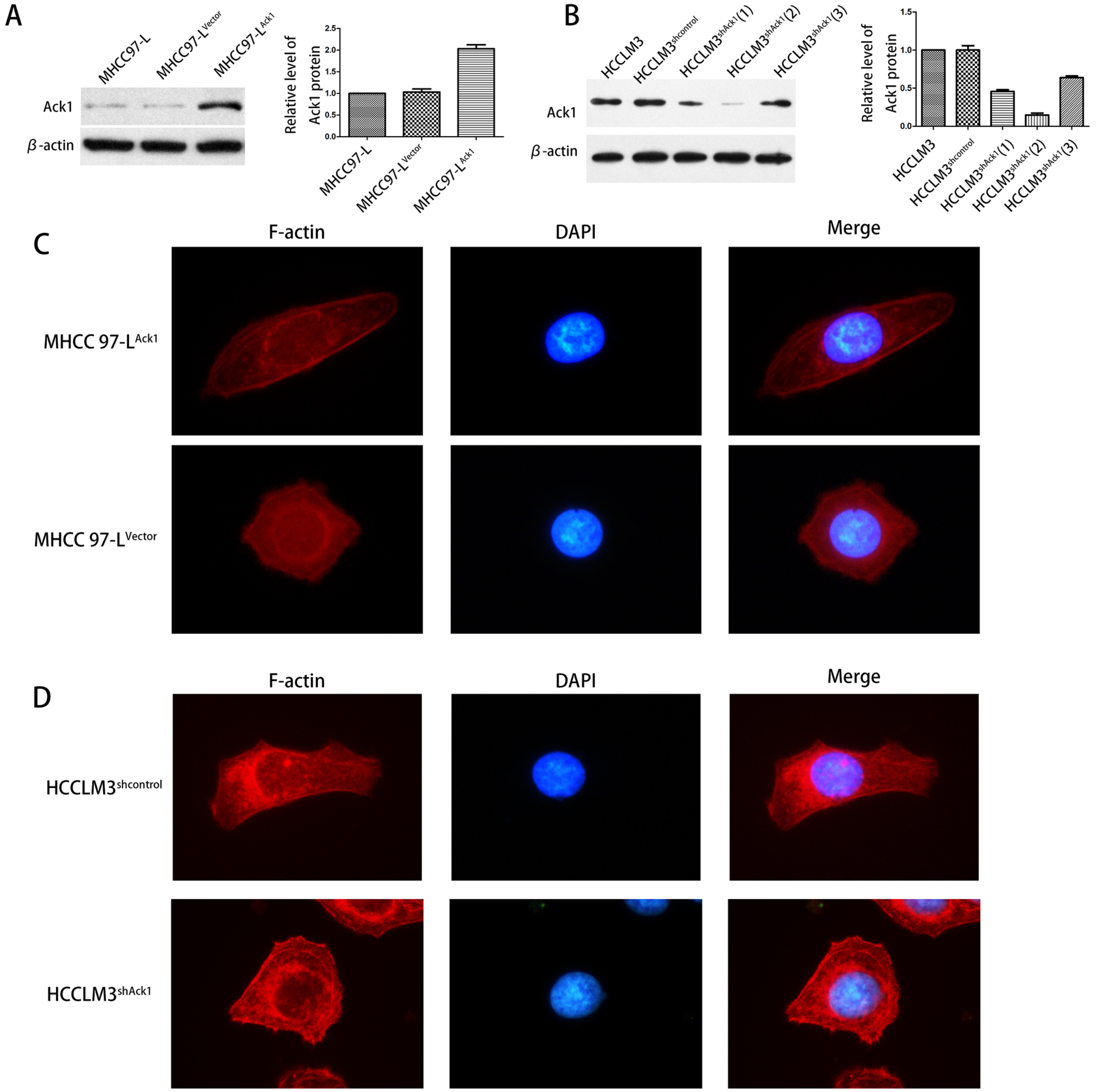

Supplementary Figure S3: Ack1 affects the cellular morphology of HCC cells. A. Ack1-expression plasmid pCMV-Tag2B-Ack1 upregulates MHCC97-L Ack1 expression. pCMV-Tag2B plasmid containing the open reading frames of human Ack1 cDNA amplified by PCR is named as pCMV-Tag2B-Ack1 and applied to transfect MHCC97-L cells using FuGENE 6 (called MHCC97-L^{Ack}). pCMV-Tag2B vector without any insert is used as the blank control for transfecting MHCC97-L cells (MHCC97-L^{Vector}). The transfected cells were selected with G418. Western blot was employed to evaluate the upregulation of exogenous Ack1 expression in MHCC97-L cells. **B.** Knockdown of Ack1 expression via shAck1 plasmid in HCCLM3 cells. Western blot was used to determine the inhibitory efficiency. Results showed that shRNA sequence 2 had the highest inhibitory efficiency (85%) of the three candidate sequences. The plasmid containing shRNA sequence 2 is named shAck1 and used for subsequent experiments. The vector containing 21bp of random sequence without targeting any known gene is named shcontrol and used as the control. **C. & D.** Representative Immunofluorescence (IF) images showed that Ack1 affected the cellular morphology of HCC cells. Cell nucleus were stained with 4′,6-diamidino-2-phenylindole (blue), and cytoskeleton was stained with Rhodamine-conjugated phalloidin. Original magnification: 400 ×.

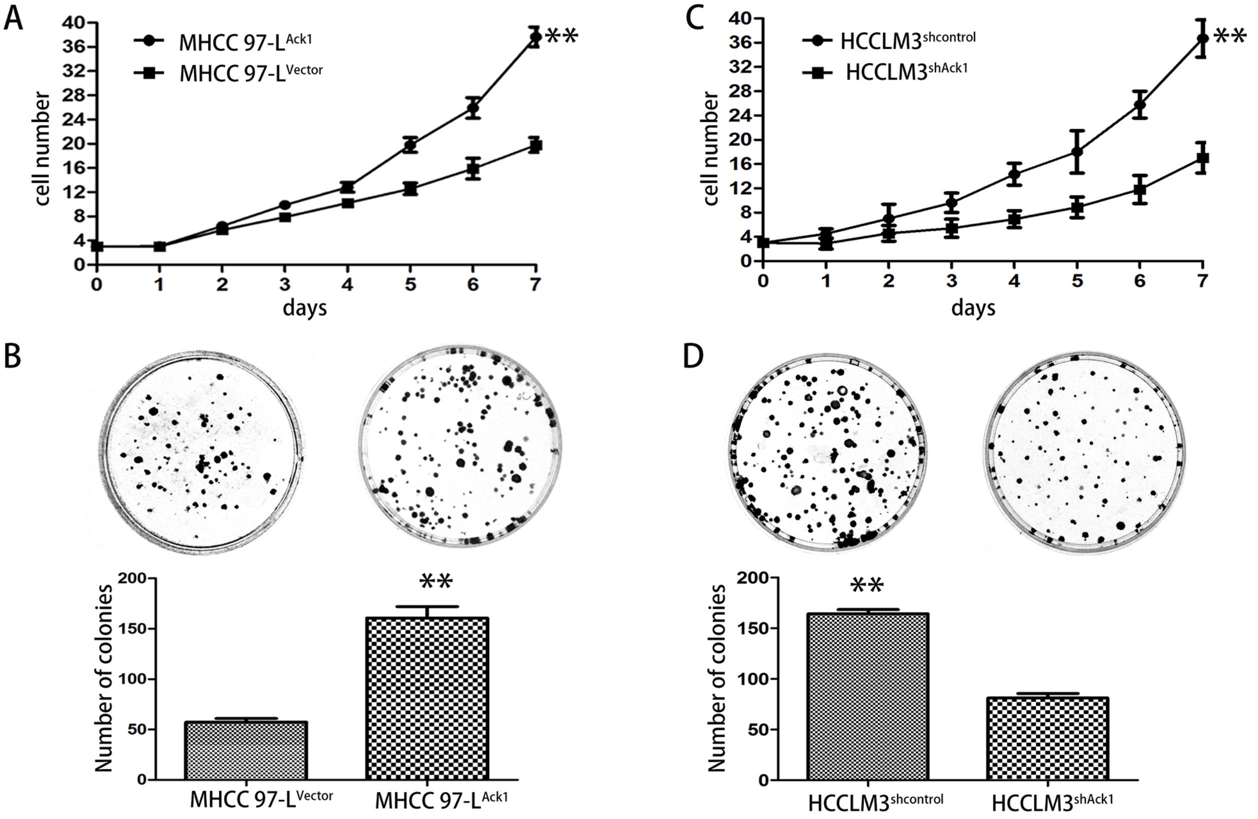

Supplementary Figure S4: Ack1 promotes HCC cells proliferation *in vitro*. A. & C. The proliferation of MHCC97-H cells with Ack1 overexpression or vector control (A) and HCCLM3 cells with Ack1 knockdown (HCCLM3^{shAck1}) or control (HCCLM3^{shcontrol}) (C) was determined as described in Materials and Methods. **B. & D.** The colony formation assays of MHCC97-L cells with Ack1 overexpress or vector control (B) and HCCLM3 cells with Ack1 knockdown (HCCLM3^{shAck1}) or control (HCCLM3^{shcontrol}) (D) were determined. The number of colony formation was counted and compared. **: $P < 0.01$.

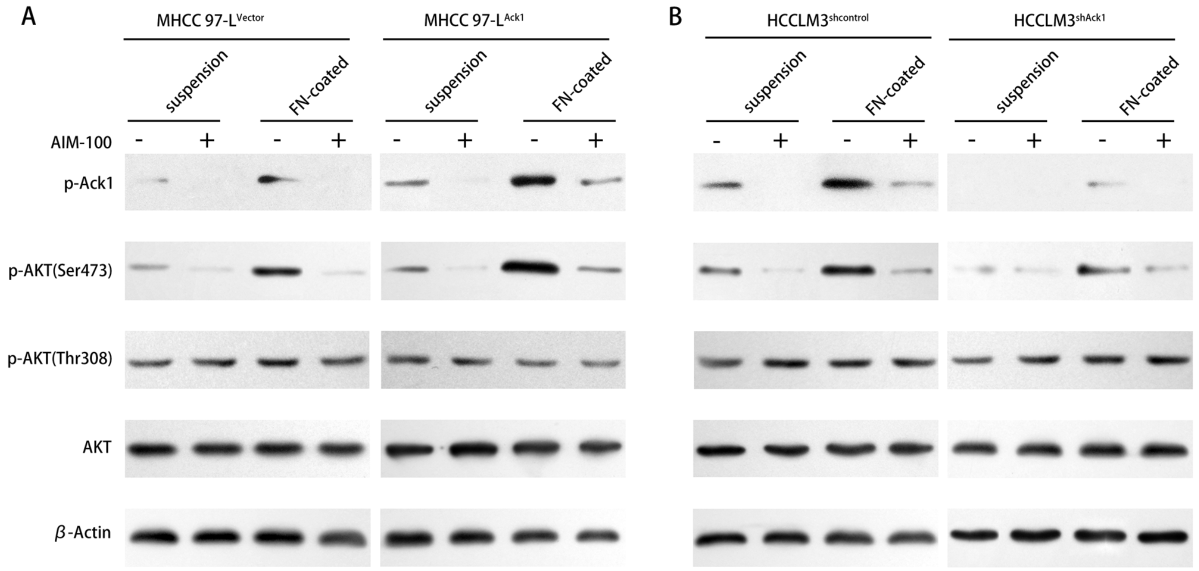

Supplementary Figure S5: Ack1 Affects Ser473-phosphorylation of AKT, but not Thr308-phosphorylation in HCC Cells. A. & B. HCC cells were either untreated or pretreated with AIM-100, a Ack1-specific inhibitor, in cell adhesion assays. Upon AIM-100 treatment, significant loss of p-Ack1 level and a concomitant decrease in Ser473-phosphorylation of AKT level was observed, whereas the effect on Thr308-phosphorylation of AKT levels was not altered.

Supplementary Table S1: Patient demographics and clinicopathological characteristics of three cohorts

Clinicopathologic variables	Training cohort		Internal validation cohort		External validation cohort	
	n	%	*n*	%	*n*	%
**Gender**						
Female	12	15.8	23	16.3	11	15.7
Male	64	84.2	118	83.7	59	84.3
**Age (years)**						
≤ 60	45	59.2	80	56.7	39	55.7
> 60	31	40.8	61	43.3	31	44.3
**HBsAg**						
Positive	68	89.5	130	92.2	62	88.6
Negative	8	10.5	11	7.8	8	11.4
**Liver cirrhosis**						
Presence	66	86.8	122	86.5	60	85.7
Absence	10	13.2	19	13.5	10	14.3
**AFP**						
≤ 20 μg/L	20	26.3	39	27.7	18	25.7
> 20 μg/L	56	73.7	102	72.3	52	74.3
**TBil(μmol/L)**						
≤ 17.1	46	60.5	84	59.6	41	58.5
> 17.1	30	39.5	57	40.4	29	41.4
**Albumin(g/L)**						
≤ 35	38	50.0	69	48.9	37	52.9
> 35	38	50.0	72	51.1	33	47.1
**Tumor number**						
Solitary	52	68.4	98	69.5	47	67.1
Multiple	24	31.6	43	30.5	23	32.9
**Tumor size**						
≤ 5 cm	42	55.3	79	56.0	39	55.7
> 5 cm	34	44.7	62	44.0	31	44.3
**Edmondson-Steiner grade**						
Low grade (I and II)	49	64.5	92	65.2	45	64.3
High grade (III and IV)	27	35.5	49	34.8	25	35.7
**Vascular invasion**						
Presence	33	43.4	81	57.4	40	57.1
Absence	43	56.6	60	42.6	30	42.9

(*Continued*)

Clinicopathologic variables	Training cohort		Internal validation cohort		External validation cohort	
	n	%	*n*	%	*n*	%
**Satellite nodules**						
Presence	27	35.5	52	36.9	32	45.7
Absence	49	64.5	89	63.1	38	54.3
**Capsular formation**						
Presence	31	40.8	56	39.7	29	41.4
Absence	45	59.2	85	60.3	41	58.6
**TNM stage**						
I	32	42.1	54	38.3	28	40.0
II–III	44	57.9	87	61.7	42	60.0
**BCLC stage**						
A	28	36.8	51	36.2	27	38.6
B/C	48	63.2	90	63.8	43	61.4

Supplementary Table S2: Correlations between Ack1 expression and clinicopathological data of HCC in external validation cohort

Clinicopathologic variable	*n*	Ack1 expression levels		*P*
		Low	High	
**Gender**				
Female	11	5	6	
Male	59	22	37	0.862
**Age (years)**				
≤60	39	15	24	
>60	31	12	19	0.983
**HBsAg**				
Positive	62	23	39	
Negative	8	4	4	0.749
**Liver cirrhosis**				
Presence	60	21	39	
Absence	10	6	4	0.249
**AFP**				
≤20 μg/L	18	8	10	
>20 μg/L	52	19	33	0.553
**TBil(μmol/L)**				
≤17.1	41	16	25	
>17.1	29	11	18	0.926

(*Continued*)

Clinicopathologic variable	*n*	Ack1 expression levels		*P*
		Low	High	
**albumin(g/L)**				
≤35	37	15	22	
>35	33	12	21	0.720
**Tumor number**				
Solitary	47	23	24	
Multiple	23	4	19	**0.011**
**Tumor size**				
≤5 cm	39	13	26	
>5 cm	31	14	17	0.313
**Edmondson-Steiner grade**				
Low grade (I and II)	45	22	23	
High grade (III and IV)	25	5	20	**0.017**
**Vascular invasion**				
Presence	40	10	30	
Absence	30	17	13	**0.007**
**Satellite nodules**				
Presence	32	7	25	
Absence	38	20	18	**0.008**
**Capsular formation**				
Presence	29	9	20	
Absence	41	18	23	0.276
**TNM Stage**				
I	28	16	12	
II–III	42	10	32	**0.005**
**BCLC Stage**				
0/A	27	14	13	
B/C	43	13	29	0.083

Supplementary Table S3: Univariable and multivariable analysis of disease-free survival (DFS) and clinicopathologic variables of HCC in external validation cohort

Clinicopathologic variables	*n*	Univariable analysis		Multivariable analysis	
		HR* (95% CI)	*P*	HR* (95% CI)	*P*
**Gender**					
Female	11	Reference			
Male	59	0.975(0.782−1.793)	0.418		NA
**Age (years)**					
≤60	39	Reference			
>60	31	1.147(0.952−1.819)	0.326		NA
**HBsAg**					
Positive	62	Reference		Reference	
Negative	8	0.771(0.502−1.164)	0. 081	0.935(0.714−1.051)	0.072
**Liver cirrhosis**					
Absence	60	Reference			
Presence	10	1.261(0.896−1.385)	0.175		NA
**AFP**					
≤20 μg/L	18	Reference		Reference	
>20 μg/L	52	1.273(1.018−1.845)	0.016	1.150(0.874−1.703)	0.079
**TBil(μmol/L)**					
≤17.1	41	Reference			
>17.1	29	1.029(0.625−1.439)	0.271		NA
**albumin(g/L)**					
≤35	37	Reference			
>35	33	1.203(0.782−2.472)	0.201		NA
**Tumor number**					
Solitary	47	Reference		Reference	
Multiple	23	1.720(1.461−2.702)	**0.009**	1.600(1.327−2.613)	**0.049**
**Tumor size**					
≤5 cm	39	Reference		Reference	
>5 cm	31	1. 857(1.601−2.284)	**0.022**	1.483(1.135−2.029)	0.261
**Edmondson − Steiner grade**					
Low grade (I and II)	45	Reference			
High grade (III and IV)	25	1.070 (0.908−1.729)	0.104		NA
**Vascular invasion**					
Absence	30	Reference		Reference	
Presence	40	5.367(2.219−10.533)	**<0.001**	3.706(2.219−9.265)	**0.015**
**Satellite nodules**					

(*Continued*)

Clinicopathologic variables	*n*	Univariable analysis		Multivariable analysis	
		HR* (95% CI)	*P*	HR* (95% CI)	*P*
Absence	38	Reference		Reference	
Presence	32	2.194(1.436–6.207)	**0.017**	1.872(1.704–5.489)	**0.023**
**Capsular formation**					
Presence	29	Reference			
Absence	41	1.073(0.749–1.092)	0.116		NA
**TNM Stage**					
I	28	Reference		Reference	
II-III	42	3.290(2.532–7.061)	**0.001**	2.701(2. 304–4. 812)	**0.002**
**BCLC Stage**					
A	27	Reference		Reference	
B/C	43	2.293(1.610–3.087)	**0.019**	1.192(1.018–2.875)	**0.012**
**Ack1 expression**					
Low	23	Reference		Reference	
High	47	4.006(2.185–7.035)	**0.035**	3.954(2.081–6.740)	**0.022**

*HR: hazard ratio

Supplementary Table S4: Univariable and multivariable analysis of overall survival (OS) and clinicopathologic variables of HCC in external validation cohort

Clinicopathologic variables	*n*	Univariable analysis		Multivariable analysis	
		HR* (95% CI)	*P*	HR* (95% CI)	*P*
**Gender**					
Female	11	Reference			
Male	59	0.976(0.852–1.533)	0.415		NA
**Age (years)**					
≤60	39	Reference			
>60	31	0.897(0.783–1.104)	0.161		NA
**HBsAg**					
Positive	62	Reference		Reference	
Negative	8	0.654(0.624–1.231)	0.047	1.004(0.739–1.341)	0.081
**Liver cirrhosis**					
Absence	60	Reference			
Presence	10	1.283(0.802–1.576)	0.126		NA
**AFP**					
≤20 μg/L	18	Reference		Reference	
>20 μg/L	52	1. 103 (0.913–1.542)	0.078	1.013(0.951–1.254)	0.054

(*Continued*)

Clinicopathologic variables	*n*	Univariable analysis		Multivariable analysis	
		HR* (95% CI)	*P*	HR* (95% CI)	*P*
**TBil(μmol/L)**					
≤17.1	41	Reference			
>17.1	29	1.216(0.871–1.562)	0.359		NA
**albumin(g/L)**					
≤35	37	Reference			
>35	33	0. 749(0.695–1.548)	0.143		NA
**Tumor number**					
Solitary	47	Reference		Reference	
Multiple	23	1.358 (1.124–3.471)	**0.014**	2.302(1.721–3.627)	**0.023**
**Tumor size**					
≤5 cm	39	Reference			
>5 cm	31	1.174(0.930–2.125)	0.172		NA
**Edmondson – Steiner grade**					
Low grade (I and II)	45	Reference		Reference	
High grade (III and IV)	25	1.210(0.967–2.409)	0.094	1.067(0.915–1.486)	0.103
**Vascular invasion**					
Absence	30	Reference		Reference	
Presence	40	2.633(1.872–4.689)	**0.005**	2.152(1.742–3.728)	**0.027**
**Satellite nodules**					
Absence	38	Reference		Reference	
Presence	32	2.324(1.731–2.927)	**0.018**	2.015(1.623–2.526)	**0.036**
**Capsular formation**					
Presence	29	Reference			
Absence	41	1.035(0. 694–1.124)	0.204		NA
**TNM Stage**					
**I**	28	Reference		Reference	
**II–III**	42	1.817(1.366–3.241)	**0.001**	1.732(1.434–2.942)	**0.021**
**BCLC Stage**					
A	27	Reference		Reference	
B/C	43	2.212(1.160–2.874)	**0.003**	1.721(1.168–2. 497)	**0.033**
**Ack1 expression**					
Low	23	Reference		Reference	
High	47	3.843(2. 729–5.891)	**<0.001**	2.461 (1.921–4.036)	**0.001**

*HR: hazard ratio
